# EGFRvIII-driven microenvironmental fibroblast activation and transformation accelerate oral cancer progression via lipocalin-2/STAT3 axis

**DOI:** 10.1016/j.neo.2025.101193

**Published:** 2025-06-04

**Authors:** Hsuan-Yu Peng, Kwang-Yu Chang, Wei-Min Chang, Chia-Yu Wu, Hsin-Lun Lee, Yung-Chieh Chang, Ko-Jiunn Liu, Shine-Gwo Shiah, Ching-Chuan Kuo, Jang-Yang Chang

**Affiliations:** a TMU Research Center of Cancer Translational Medicine, Taipei Medical University Hospital, College of Medicine, Taipei Medical University, Taipei, 110301 Taiwan, ROC; b National Institute of Cancer Research, National Health Research Institutes, Tainan, 70456, Taiwan, ROC; c Division of Hematology/Oncology, Department of Internal Medicine, National Cheng Kung University Hospital, College of Medicine, National Cheng Kung University, Tainan, Taiwan, ROC; d School of Oral Hygiene, College of Oral Medicine, Taipei Medical University, Taipei, 110301, Taiwan, ROC; e Research Center of Oral Translational Medicine, College of Oral Medicine, Taipei Medical University, Taipei, 110301, Taiwan, ROC; f Division of Oral and Maxillofacial Surgery, Department of Dentistry, Taipei Medical University Hospital, Taipei, 110301, Taiwan, ROC; g School of Dental Technology, College of Oral Medicine, Taipei Medical University, Taipei, 110301, Taiwan, ROC; h Department of Radiology, School of Medicine, College of Medicine, Taipei Medical University, Taipei, 110301, Taiwan, ROC; i Graduate Institute of Clinical Medicine, College of Medicine, Taipei Medical University, Taipei, 110301, Taiwan, ROC; j Department of Radiation Oncology, Taipei Medical University Hospital, Taipei, 110301, Taiwan, ROC; k TMU Proton Center, Taipei Medical University, Taipei, 110301, Taiwan, ROC; l National Institute of Cancer Research, National Health Research Institutes, No. 35 Keyan Road, Zhunan Town, Miaoli County, 35053, Taiwan, ROC; m Institute of Biotechnology and Pharmaceutical Research, National Health Research Institutes, Miaoli County, 35053, Taiwan, ROC; n Graduate Institute of Biomedical Sciences, China Medical University, Taichung, 404, Taiwan, ROC

**Keywords:** EGFRvIII, Oral squamous cell carcinoma, Fibroblast activation, Tumor microenvironment, Lipocalin-2, STAT3 signaling

## Abstract

Oral squamous cell carcinoma (OSCC) is an aggressive malignancy frequently characterized by dysregulated epidermal growth factor receptor (EGFR) signaling. Among EGFR mutation, EGFRvIII, an extracellular domain truncated form without exons 2–7, exhibits ligand-independent and constitutive EGFR activation. Although EGFRvIII functions as an oncogene in glioblastoma, its role in OSCC remains unclear. Here, we demonstrate that EGFRvIII is highly prevalent in OSCC, with approximately 70 % of OSCC tumor samples revealing high EGFRvIII expression. EGFRvIII enhances metastatic and proliferative potential, while its knockdown significantly reduces these malignant phenotypes. Beyond its direct oncogenic effects, EGFRvIII actively remodels the tumor microenvironment (TME) by recruiting and activating fibroblasts. In both xenograft models and co-culture systems, OSCC cells expressing EGFRvIII stimulated the expression of fibroblast activation markers—including α-smooth muscle actin (α-SMA), platelet-derived growth factor receptors (PDGFRA/PDGFRB), and collagen—thereby promoting a tumor-supportive stroma. Moreover, RNA sequencing and cytokine array analyses revealed that EGFRvIII induces lipocalin-2 (LCN2) expression and secretion. Elevated LCN2 in the conditioned medium from OSCC-EGFRvIII cells further stimulates fibroblast activation via the STAT3 signaling pathway, as pharmacological inhibition of STAT3 attenuates LCN2-driven fibroblast activation. Furthermore, exposure to environmental carcinogens such as nicotine-derived nitrosamine ketone (NNK) and arecoline enhances EGFRvIII expression and downstream signaling, exacerbating tumor aggressiveness. These findings reveal a positive feedback loop in which EGFRvIII fosters OSCC progression by stimulating LCN2-STAT3-mediated fibroblast activation. Targeting EGFRvIII and its downstream effectors may therefore represent a promising strategy to mitigate OSCC progression and improve therapeutic outcomes.

## Highlights

• EGFRvIII is highly prevalent in OSCC and drives tumor cell proliferation, migration, and invasion.
• EGFRvIII remodels the tumor microenvironment by recruiting and activating CAFs.
• LCN2 mediates EGFRvIII-driven fibroblast activation via STAT3 signaling.
• NNK and arecoline upregulate EGFRvIII and its downstream oncogenic signaling.
• Targeting the EGFRvIII-LCN2-STAT3 axis may suppress tumor progression and improve OSCC therapy.

## Introduction

Oral squamous cell carcinoma (OSCC) is the most common malignant tumor of the oral cavity, accounting for over 90 % of oral cancers and constituting a significant subset of head and neck squamous cell carcinoma (HNSCC) [[1], [2], [3]]. It is a global health concern due to its high incidence, aggressive clinical behavior, and poor prognosis, particularly in advanced stages. Despite advancements in surgical techniques, chemotherapy, and radiotherapy, the 5-year survival rate for OSCC patients remains low, especially for those diagnosed at late stages or with metastasis [[4], [5], [6], [7]]. Among the key drivers of OSCC progression, the overexpression of epidermal growth factor receptor (EGFR) has been strongly linked to enhanced tumor growth, invasion, and resistance to therapy [[8], [9], [10], [11]]. EGFR, a transmembrane tyrosine kinase receptor, plays a pivotal role in regulating cell proliferation, differentiation, and survival, making it an important therapeutic target in multiple cancers, including HNSCC [12]. Although EGFR-targeted therapies, such as tyrosine kinase inhibitors (TKIs) and monoclonal antibodies, have shown promise, their clinical efficacy in advanced head and neck cancers—including OSCC—is often compromised by resistance mechanisms [13]. One such mechanism involves a specific EGFR mutation, EGFRvIII, characterized by the deletion of exons 2–7. This alteration leads to ligand-independent and constitutive activation of EGFR, which drives oncogenic signaling and contributes to therapy resistance [[14], [15], [16]]. Notably, EGFRvIII has been extensively characterized in glioblastoma, where it is associated with aggressive tumor behavior and reduced responses to EGFR-targeted therapies [17]. In our previous analysis of 108 surgically resected OSCC samples, EGFRvIII expression was significantly correlated with patient survival, suggesting its potential as a critical driver of OSCC progression and a promising therapeutic target [18]. However, the overall prevalence and functional role of EGFRvIII in OSCC remain incompletely understood, necessitating further investigation. Tumor progression is driven by both tumor-intrinsic factors and complex interactions within the tumor microenvironment (TME) [19,20]. The TME, composed of fibroblasts, immune cells, and extracellular matrix components, plays a pivotal role in promoting tumor growth, invasion, and resistance to therapy [21,22]. Understanding how EGFRvIII influences the TME, particularly in fibroblast activation and stromal remodeling, could uncover novel therapeutic strategies for OSCC. Beyond its oncogenic role within tumor cells, EGFRvIII profoundly impacts the TME. In glioblastoma, EGFRvIII enhances tumor heterogeneity, therapy resistance, and progression by inducing cytokine secretion, immune evasion, and cancer-associated fibroblast (CAF) activation [[23], [24], [25]]. Activated CAFs exhibit myofibroblast-like properties, including contractile stress fiber formation and α-smooth muscle actin (α-SMA) expression, which contribute to tumor desmoplasia [26,27]. In OSCC, CAFs play a critical role in tumor progression by secreting cytokines, remodeling the extracellular matrix, and supporting immune evasion [[28], [29], [30], [31]]. However, the mechanisms through which EGFRvIII drives CAF activation and recruitment in OSCC remain poorly understood, warranting further investigation.

Lipocalin-2 (LCN2), also known as neutrophil gelatinase-associated lipocalin (NGAL), is a secreted glycoprotein involved in iron homeostasis and immune modulation, and it has emerged as a key mediator of tumor progression in various cancers [32,33]. In cancer cells, EGFRvIII has been shown to induce LCN2 expression, thereby promoting tumor cell invasion, immune suppression, and the recruitment of immunosuppressive cell types [34]. LCN2 is also implicated in extracellular matrix (ECM) remodeling and metastasis in breast and esophageal cancers [35,36], where elevated LCN2 levels correlate with enhanced migratory and invasive capacities of tumor cells. Furthermore, LCN2 expression is abundant in neutrophils and in a distinct subpopulation of fibroblasts [37], suggesting that its role in modulating the TME extends beyond cancer cells. Nevertheless, the exact connection between EGFRvIII-mediated LCN2 induction and its downstream effects on CAF activation remains to be elucidated. Emerging evidence indicates that LCN2 can influence fibroblast behavior [35], implying a potential axis for EGFRvIII-driven TME reprogramming in OSCC. Thus, it is worth to further elucidating this putative EGFRvIII–LCN2–CAF signaling loop and determining whether contributes to OSCC progression and therapy resistance.

Additionally, environmental carcinogens such as nicotine-derived nitrosamine ketone (NNK) and arecoline have been strongly associated with OSCC initiation and progression [38,39]. These agents contribute to chronic inflammation, genetic mutations, and remodeling of the TME, including CAF recruitment and activation [38,39]. However, their impact on EGFRvIII expression and associated oncogenic pathways in OSCC has not been fully characterized. This study aims to elucidate the molecular mechanisms through which EGFRvIII drives OSCC progression and modulates the TME. Specifically, we examine EGFRvIII-regulated pathways and their downstream effects on CAF activation, focusing on the roles of LCN2 and STAT3 signaling. Additionally, we explore how environmental carcinogens, such as NNK and arecoline, influence EGFRvIII expression and its functional consequences. These findings aim to provide novel insights into the multifaceted role of EGFRvIII in OSCC and highlight its potential as a therapeutic target for disrupting tumor-stroma interactions.

## Materials and methods

### Cell lines

Human Dysplastic Oral Keratinocyte (DOK) cells (RRID: CVCL_1180, a gift from Dr. Shine-Gwo Shiah, National Health Research Institutes, Taiwan), Oral mucosal fibroblasts (OMFs) (a gift from Dr Chin-Wei Wang, Division of Periodontics, Department of Dentistry, Taipei Medical University Hospital, Taipei) and human oral cancer Cal-27 cells (Cat# CRL-2095, RRID: CVCL_1107) were obtained from ATCC (Rockville, MD, USA) and cultured in DMEM. HSC-2 (Cat# JCRB0622, RRID: CVCL_1287), HSC-3 (Cat# JCRB0623, RRID: CVCL_1288), HSC-3-M3 (Cat# JCRB1354, RRID: CVCL_8323), and HSC-4 (Cat# JCRB0624, RRID: CVCL_1289) cells were obtained from JCRB (Tokyo, Japan) and maintained in α-MEM (Gibco, Grand Island, NY, USA). SAS cells (Cat# JCRB0620, RRID: CVCL_1675) were also purchased from JCRB and cultured in high-glucose DMEM (Gibco). SCC-4 (Cat# CRL-1624, RRID: CVCL_1684), SCC-9 (Cat# CRL-1629, RRID: CVCL_1685), SCC-15 (Cat# CRL-1623, RRID: CVCL_1681), and SCC-25 (Cat# CRL-1628, RRID: CVCL_1681) cells were cultured in DMEM/F12 (1:1) supplemented with 1 mM sodium pyruvate and 400 nM hydrocortisone. Human embryonic kidney 293T cells (RRID: CVCL_0063) were obtained from ATCC (Rockville, MD, USA). All media were supplemented with 10 % fetal bovine serum, 1 % penicillin-streptomycin (PS), and 1 % nonessential amino acids (Gibco, Grand Island, NY, USA). All cells were cultured at 37 °C in a 5 % CO2 atmosphere and maintained within 3 months of resuscitation from the frozen aliquots with <20 passages for each experiment. The cell lines were authenticated by morphology or by Short Tandem Repeat (STR) analysis by analyzing multiple locations within the genome containing short DNA sequence repeats. The resulting DNA profiles were used to confirm the identity and purity of all cell lines through comparison to the STR reference database in the past 3 years. In addition, all cells were regularly checked for mycoplasma infection and cell morphology to keep cells healthy.

### Analysis of the expression of the EGFRvIII isoform

Human Oral tissue samples were obtained from patients at the Taipei Medical University Hospital (TUH) with the understanding and written consent of each subject. The study was approved by the Taipei Medical University Hospital (TUH) Institutional Review Board (approval number: N202204007). The study methodologies conformed to the standards set by the Declaration of Helsinki. Total RNA was isolated from tissue samples using the RNeasy Micro Kit (Cat# 74004, QIAGEN) following the manufacturer’s instructions. The RNA quantity and purity were determined by measuring the A260/280 absorbance ratio using a NanoDrop spectrophotometer (BRED-1000, Blue-Ray).

EGFR amplicons are frequently mutated, with variant 3 (EGFRvIII), characterized by the deletion of exons 2 to 7, being the most common. To detect the presence of EGFRvIII, 100 ng of total RNA was reverse-transcribed into first-strand cDNA using the RevertAid RT Reverse Transcription Kit (Cat# K1691, Thermo Scientific) following the manufacturer’s protocol. The region spanning EGFR exon 1 to exon 8 was amplified by PCR using the following primers: forward 5′-GGGCTCTGGAGGAAAAGAAA-3′ and reverse 5′-AGGCCCTTCGCACTTCTTAC-3′. The PCR amplification was performed under the following conditions: an initial denaturation at 95°C for 2 min, followed by 30 cycles of 95°C for 30 s, 55°C for 45 s, and 72°C for 1 min and 30 s, with a final extension step at 72°C for 5 min. RT-PCR products were visualized by 0.8 % agarose gel electrophoresis, where a 128 bp product indicated the presence of EGFRvIII, and a 929 bp product corresponded to wild-type (WT) EGFR.

MSCV-XZ066-EGFRvIII (Plasmid #20737), which expresses the EGFRvIII variant, was amplified and used as a positive control to validate the presence of the EGFRvIII-specific fragment. The protocol and primer set used in this study were based on previously published work [16].

### Plasmids and transfection

The Pcmv6-EGFRvIII plasmid was constructed by inserting the full-length cDNA of EGFRvIII (NM_001346941.2) into the vector. For EGFRvIII knockdown, HSC-2, HSC-3, and SCC-9 oral squamous cell carcinoma (OSCC) cells were transfected with an siEGFRvIII (GeneID: 1956; Cat# abx941278, Abbexa, Cambridge, UK) using the TransIT-X2 Transfection Reagent (Mirus Bio, Madison, WI, USA), according to the manufacturer’s protocol. For LCN2 knockdown, OMFs were transfected with an siLCN2 (GeneID: 3934; Cat# l-003679-00-0005, Dharmacon, Lafayette, CO, USA) using the same TransIT-X2 transfection protocol.

For lentivirus production, 293T cells were co-transfected with the plasmids pMD.G, pCMV▵R8.91, and lentiviral transfer plasmids using the TransIT-LT1 Transfection Reagent (Mirus, Madison, WI, USA). Culture medium containing lentivirus was harvested 48 h post-transfection. The medium was centrifuged at 500 × g for 5 min to remove cells and filtered through a 0.45-μm filter. The lentiviral stocks were supplemented with polybrene (8 μg/mL) and used to infect target cells for 6 h to ensure efficient transduction.

### Transwell migration and invasion assays

Transwell migration and invasion assays were conducted using Transwell insert plates (PET membrane, 8-μm pore size; SPL 36224, Korea). For the invasion assay, the insert membranes were pre-coated with 2 mg/mL Matrigel (Corning Matrigel Membrane Matrix, Cat# 356234) at 37°C for 1 h, whereas the migration assay was performed without coating.

OSCC Cell Migration and Invasion Assays:

For the migration assay, OSCC cells (5 × 10⁴ cells per well) were suspended in serum-free medium and seeded into the upper chamber, with 600 μL of medium containing 2 % FBS added to the lower chamber as a chemoattractant. For the invasion assay, OSCC cells (2 × 10⁵ cells per well) were seeded into the Matrigel-coated upper chamber under the same conditions.

OMF Migration Assay:

Oral mucosal fibroblasts (OMFs; 2 × 10⁴ cells per well) pre-treated with LCN2 (100 ng/mL) were seeded into the upper chamber in serum-free medium, while 600 μL of medium containing 2 % FBS was added to the lower chamber. Cells were allowed to migrate for 24 h.

Cancer Cell Migration Assay Co-cultured with OMFs:

OSCC cells (5 × 10⁴ cells per well) were suspended in serum-free medium and seeded into the upper chamber, OMFs (1 × 10⁵ cells per well) were seeded into the lower chamber containing 600 μL of medium. OSCC cells were then seeded into the upper chamber, and the system was incubated for 24 h to allow for cell invasion.

After the incubation period, migrated and invaded cells were fixed with 100 % ethanol at 4°C for 60 min, washed twice with PBS, and the interiors of the inserts were carefully wiped clean with wet cotton swabs. The membranes were stained with 0.1 % crystal violet in 20 % methanol for 15 min and washed with PBS to remove excess stain. Cells attached to the membranes were visualized under an inverted microscope at 200 × magnification (Olympus Corporation, Hachioji, Tokyo, Japan). Images were captured and the number of migrated or invaded cells was quantified using analytical imaging software (Imaging Research, Ontario, Canada).

### Cell migration assays – wound healing method

Wound healing assays were performed using Culture-Insert 3 Well in µ-Dish 35 mm, high (Cat. No: 80366, Ibidi, Thistle Scientific). The silicone inserts created three distinct wells to facilitate cell seeding and wound formation. Cells were seeded at a density of 1 × 10⁵ cells/mL, with 100 µL of cell suspension added to each well of the culture insert. After overnight incubation in complete medium to allow the formation of a confluent monolayer, cells were serum-starved for 24 h to induce quiescence. Following the removal of the silicone insert, cells were incubated at 37°C with 5 % CO₂, and images of the wound area were captured at 0, 8, and 16 h to monitor cell migration. The number of migrated or invaded cells was quantified using analytical imaging software on the EVOS M5000 Imaging System (Thermo Fisher Scientific).

### Oral mucosal fibroblasts co-cultured with cancer cells

Oral squamous cell carcinoma (OSCC) cells (1 × 10⁵ cells per well) were seeded into the upper well of a 24-well Transwell chamber (PET membrane, 0.4-μm pore size; SPL 36224, Korea) to establish a co-culture system. Oral mucosal fibroblasts (OMFs; 5 × 10⁴ cells per well) were seeded into the lower well of the same Transwell chamber. OMF growth medium was used in both compartments to support cell growth. The co-culture system was maintained for 72 h in a humidified incubator at 37°C with 5 % CO₂.

### Animal studies

All animal experiments were approved by the Institutional Animal Care and Utilization Committee (IACUC) of Taipei Medical University, Taipei, Taiwan. Mice were maintained in a specific pathogen-free (SPF) animal facility under controlled conditions at 20 ± 2°C with a 12/12-h light/dark cycle. Animals were provided with free access to water and a standard laboratory chow diet. All mice were monitored for abnormal tissue growth or signs of distress in accordance with AAALAS guidelines, and euthanasia was performed if excessive deterioration in health was observed.

To assess the effect of EGFRvIII expression on the reprogramming of fibroblasts in the tumor microenvironment, 1 × 10⁶ EGFRvIII-overexpressing or control MTCQ1 cells were implanted subcutaneously into the flanks of 6-week-old C57BL/6JNarl (B6) mice (obtained from the Taiwan National Laboratory Animal Center). On day 35 post-implantation, all mice were euthanized, and the xenograft tumors were collected for analysis. Tumor weight and size were measured and compared between groups to evaluate the impact of EGFRvIII expression.

### Immunohistochemical (IHC) staining

Immunohistochemistry was performed on formalin‐fixed, paraffin‐embedded sections using the Leica Bond RX autostainer (Leica Microsystems, Buffalo Grove, IL, USA). After dewaxing and rehydration, samples underwent antigen retrieval at pH6 (ER1)/pH9 (ER2) for 10–30 min, and the diluent was used for protein blocking. α‐SMA Ab (ER2 30 min, 1 : 200, catalog no NB600‐531), PDGFRA Ab (ER2 20 min 1 : 200, catalog no NBP2-67025; Novus Biologicals, Abingdon, Oxon, UK), PDGFRB Ab (ER2 20 min, 1 : 200, catalog no ab32570), Vimentin (ER1 20 min 1 :200, catalog no ab92547; Abcam), Collagen I Ab (ER1 20 min 1 :100, catalog no IR292-962; iReal) were detected with the Bond Polymer Refine Detection (Leica Microsystems), and FAP Ab (ER1 20 min 1 : 100, catalog no ab207178; Abcam), followed by counterstaining with hematoxylin and bluing reagent (Leica Microsystems). For IHC data analysis, slides were scanned at 40 × magnification using Aperio Digital Pathology Slide Scanners; high‐power images (40 × magnification) were randomly selected and analyzed by leica aperio imagescope digital slide viewer v9.1.19.1568.

### Immunofluorescent staining

Cells were fixed with freshly prepared 4 % paraformaldehyde (Merck, Whitehouse Station, NJ) for 10 min at room temperature, followed by permeabilization with 0.05 % Triton X-100 in PBS for 5 min. After permeabilization, cells were blocked with 3 % bovine serum albumin (BSA) in PBS at 37°C for 1 h to minimize nonspecific binding. For α-SMA staining, cells were incubated overnight at 4°C with a mouse anti-α-SMA monoclonal antibody (ab7817, diluted in 3 % BSA/PBS). The following day, cells were incubated with a FITC-conjugated secondary antibody (ab7064, 1:500, Abcam) at room temperature for 2 h in the dark. For rabbit anti-Vimentin monoclonal antibody and rabbit anti-TGFβ1 monoclonal antibody staining, cells were sequentially incubated with Vimentin antibody (IR45-137, 1:1000) or Lipocalin-2/NGAL antibody ((26991-1-AP), 1:200) at room temperature for 3.5 h. This was followed by incubation with a Texas Red-conjugated secondary antibody (ab7088, 1:500, Abcam) at room temperature for 2 h in the dark. Finally, nuclei were counterstained with 4′,6-diamidino-2-phenylindole (DAPI) at room temperature for 5 min. Coverslips were mounted with ProLong Gold anti-fade reagent (Invitrogen, Carlsbad, CA) to preserve fluorescence. Fluorescent images were captured using the EVOS M5000 Microscopy Workstation (Thermo Fisher Scientific).

### Statistical analysis

Except for the animal studies, all experiments were repeated at least 2 or 3 times (indicated as n or N). Data were presented as the mean ± standard deviation (SD) from repeated independent experiments. Differences between various treatment groups were assessed using the Student's t-test. Between‐group differences were considered significant at P < 0.05. Data analyses were performed using graphpad prism Ver. 8.0 (San Diego, CA, USA).

## Results

### EGFRvIII expression in OSCC and its detection

**Fig. 1:**
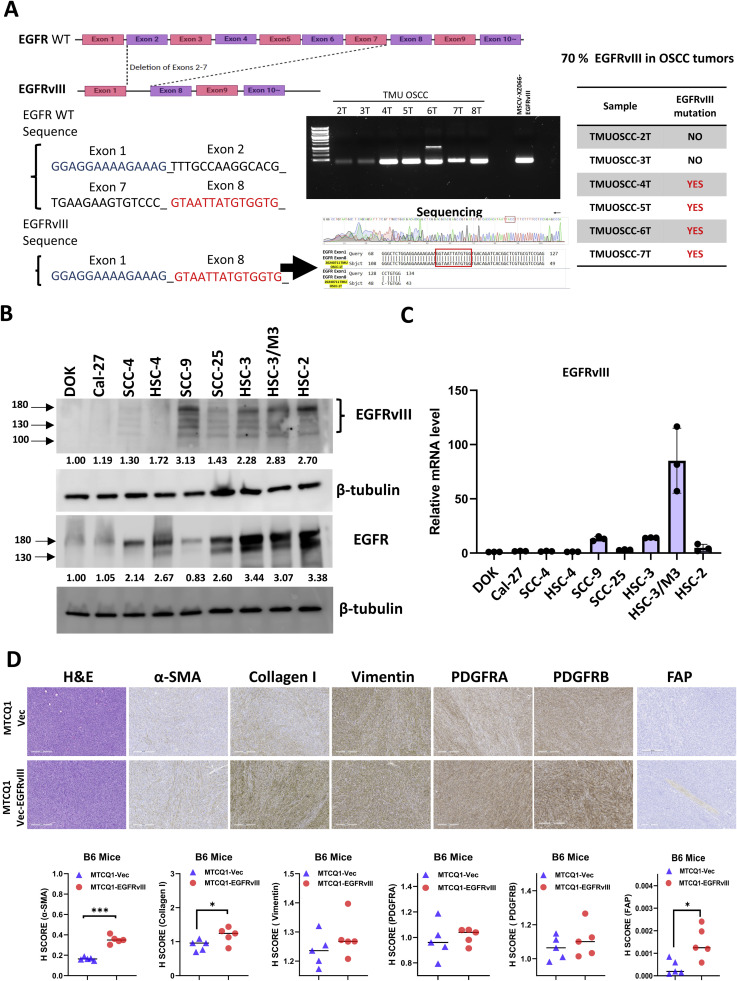
Aberrant expression of EGFRvIII in OSCC. A. Schematic structure of EGFR WT and EGFRvIII. Representative agarose gel electrophoresis images show EGFRvIII-specific PCR bands, further confirmed by Sanger sequencing chromatograms and sequence alignments. A summary table indicates EGFRvIII positivity ("YES") or negativity ("NO") across OSCC specimens, with a mutation detection rate of approximately 70 %. B. Western blot analysis of EGFRvIII protein levels in OSCC cell lines. β-tubulin served as the loading control. Protein band intensities were quantified by densitometry, normalized to β-tubulin, and presented as numerical values below the gels. C. Quantitative real-time PCR (qRT-PCR) analysis of EGFRvIII mRNA expression in OSCC cell lines. GAPDH was used as the internal normalization control. D. Xenograft tumors were established by subcutaneous injection of MTCQ1 cells (mouse OSCC cells) overexpressing EGFRvIII or a control vector into C57BL/6 mice. Tumors were harvested and analyzed on day 35 post-injection. Representative photomicrographs and dot plots of immunohistochemical (IHC) staining demonstrate the expression of fibroblast activation markers (α-SMA, Collagen I, Vimentin, FAP, PDGFRA, and PDGFRB) in tumor tissues derived from MTCQ1-EGFRvIII and control vector groups. Scale bar: 300 μm; 40 × magnification. Data are presented as mean ± SD, and statistical significance was determined using Student’s t-test (*P < 0.05; *P < 0.01; *N* = 1).

EGFRvIII is a deletion mutation of the EGFR gene, characterized by the loss of exons 2 to 7, resulting in ligand-independent, constitutive activation of EGFR [16]. We have reported previously that 75 % of head and neck cancer tissue harbored EGFRvIII mutation by immunohistological study [18]. To further confirm the presence of EGFRvIII in human OSCC, we extracted RNA from seven histologically confirmed oral tumor samples. Using RT-PCR, we detected the unique EGFRvIII sequence as outlined in the Materials and Methods. As anticipated, the 128-bp PCR product was absent in normal oral tissues, indicating that EGFRvIII is tumor-specific mutation. (**Supplemental Fig. 1A**). Five out of seven (70 %) of our OSCC specimens analyzed contained the EGFRvIII mutation, which was further validated through PCR, agarose gel electrophoresis, and Sanger sequencing (Fig. 1A). Furthermore, both Western blotting and RT-PCR analyses revealed elevated EGFRvIII expression in most OSCC cell lines compared to the human Caucasian dysplastic oral keratinocyte (DOK) line (Fig. 1B-C). Previous studies have indicated that histologically normal mucosa adjacent to OSCC can harbor cancer-associated fibroblasts (CAFs) that support local and regional tumor recurrence [40]. To examine whether EGFRvIII induces fibroblast activation in the tumor microenvironment (TME), we established xenograft models by injecting MTCQ1-EGFRvIII or MTCQ1-control cells into C57BL/6 J mice. After 35 days, tumors were harvested for analysis. Although tumor size and weight remained comparable between both groups (**Supplemental Fig. 2**), immunohistochemical (IHC) analysis revealed a significant increase in α-SMA(+), Collagen I(+), and FAP(+) fibroblasts in MTCQ1-EGFRvIII-derived tumors (Fig. 1D). These findings suggest that EGFRvIII is a prevalent, tumor-specific mutation in OSCC that modulates the TME by promoting fibroblast activation.

### EGFRvIII enhances tumor cell growth, migration, and invasion

**Fig. 2:**
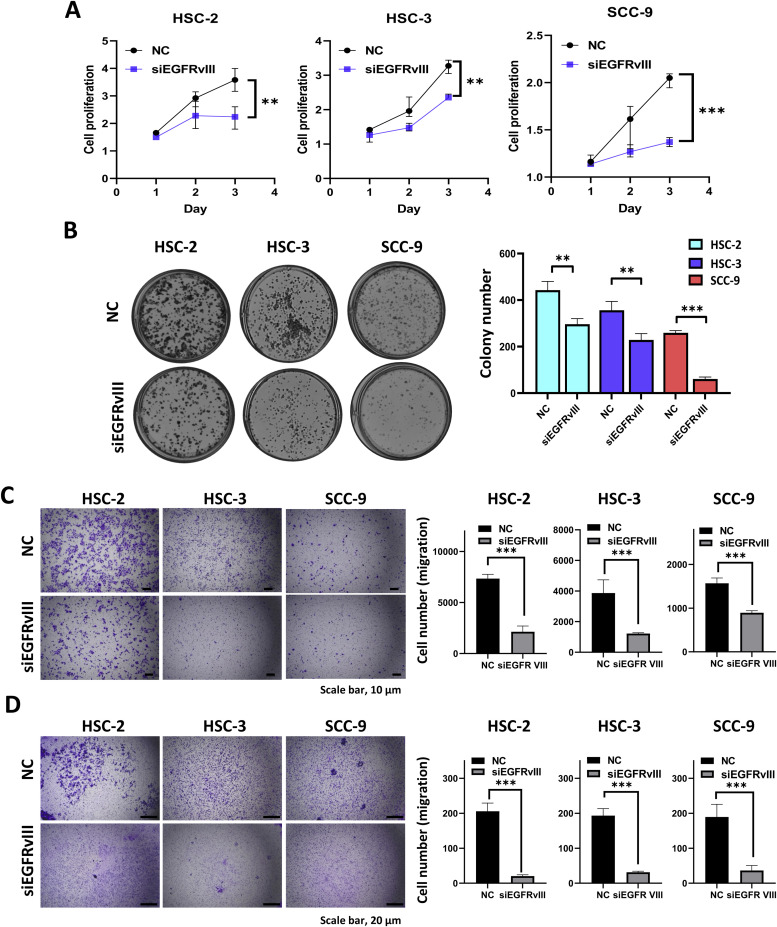
EGFRvIII knockdown inhibits OSCC growth, migration, and invasion A. Growth curves of HSC-2, HSC-3, and SCC-9 cells transfected with siEGFRvIII or non-targeting control (NC) for 72 h. Cells were seeded into 96-well plates, and CCK-8 assays were conducted at 24, 48, and 72 h post-seeding. B. Colony formation assays of HSC-2, HSC-3, and SCC-9 cells transfected with siEGFRvIII or NC for 72 h. Cells were seeded into 6-well plates and cultured for 7 days before colony counting. C. Transwell migration assays performed for 24 h using HSC-2, HSC-3, and SCC-9 cells transfected with siEGFRvIII or NC for 72 h. Scale bar, 10 μm. D. Transwell invasion assays using Matrigel-coated inserts were conducted for 24 h under the same conditions as in (C). Scale bar, 20 μm. Error bars represent the mean ± standard deviation from three independent experiments. Statistical significance was determined using appropriate tests (*p < 0.05).

To investigate the functional role of EGFRvIII knockdown in OSCC, we silenced EGFRvIII expression using siRNA in HSC-2, HSC-3, and SCC-9 cells. Compared to control cells, EGFRvIII knockdown significantly reduced growth rates in HSC-2, HSC-3, and SCC-9 cells, as assessed by CCK-8 assays (Fig. 2A). Colony formation assays demonstrated a marked decrease in both the number and size of colonies, indicating impaired clonogenic survival (Fig. 2B). Transwell migration and invasion assays were performed to evaluate the impact of EGFRvIII knockdown on OSCC cell motility. For migration assays, cells were seeded into uncoated transwell chambers and incubated for 24 h. EGFRvIII knockdown in HSC-2, HSC-3, and SCC-9 cells resulted in a significant reduction in migrated cell numbers compared to controls, suggesting diminished motility (Fig. 2C). For invasion assays, cells were seeded into Matrigel-coated transwell chambers to assess their ability to degrade and invade through an extracellular matrix (ECM) barrier. EGFRvIII knockdown significantly decreased the invasive capacity of OSCC cells, as evidenced by a reduced number of invading cells (Fig. 2D). These findings indicate that EGFRvIII knockdown impairs OSCC cell proliferation, migration, and invasion, reinforcing its role as a key driver of tumor progression. In contrast, EGFRvIII-overexpressing cells exhibited enhanced tumor cell motility and invasiveness **(Supplemental Fig. 3)**. Together, these results highlight EGFRvIII as a critical mediator of OSCC aggressiveness and emphasize its potential as a therapeutic target.

### EGFRvIII regulates MMP2 and MMP9 expression and activity in OSCC cells

**Fig. 3:**
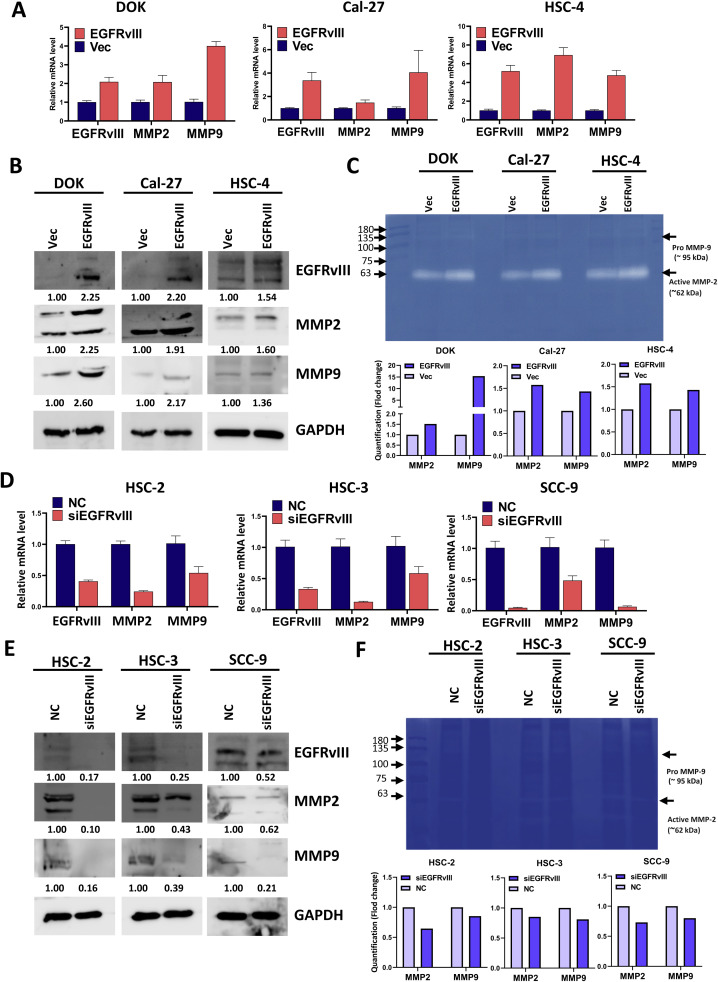
EGFRvIII regulates MMP2 and MMP9 expression and activity A. qPCR analysis of MMP2 and MMP9 mRNA levels in DOK, Cal-27, and HSC-4 cells transfected with EGFRvIII or control vector for 72 h. B. Western blot analysis of MMP2, MMP9, and EGFRvIII protein levels in DOK, Cal-27, and HSC-4 cells 72 h post-transfection. GAPDH was used as a loading control. Protein band intensities were quantified by densitometry, normalized to GAPDH, and presented as numerical values below the gels. C. Gelatin zymography for determining MMP-2 and MMP-9 activities in cell lysates from DOK, Cal-27, and HSC-4 cells transfected with EGFRvIII or control vector for 72 h. Clear bands on gelatin-containing 7.5 % polyacrylamide gels indicate gelatinolytic activities, which were quantified by densitometry. D. qPCR analysis of MMP2 and MMP9 mRNA levels in HSC-2, HSC-3, and SCC-9 cells transfected with siEGFRvIII or non-targeting control (NC) for 72 h. E. Western blot analysis of MMP2 and MMP9 protein levels in siEGFRvIII-transfected HSC-2, HSC-3, and SCC-9 cells harvested 72 h post-transfection. GAPDH was used as a loading control. Protein band intensities were quantified by densitometry, normalized to GAPDH, and presented as numerical values below the gels. F. Gelatin zymography for determining MMP-2 and MMP-9 activities in cell lysates from HSC-2, HSC-3, and SCC-9 cells transfected with siEGFRvIII or NC for 72 h.

Matrix metalloproteinases-2 and −9 (MMP-2, −9) are key enzymes involved in the degradation of the extracellular matrix, a crucial step for cellular invasion, and are known to play a significant role in the metastasis of various cancers [41,42]. To investigate the role of EGFRvIII in modulating MMP2 and MMP9 expression, we conducted qPCR, gelatin zymography, Western blot and analyses. qPCR analysis revealed that MMP2 and MMP9 mRNA levels were significantly upregulated in DOK, Cal-27, and HSC-4 cells transfected with EGFRvIII compared to control vector-transfected cells, respectively (Fig. 3A). Similarly, Western blot analysis demonstrated increased protein levels of MMP2 and MMP9 in EGFRvIII-overexpressing cells (Fig. 3B). To assess the functional impact of EGFRvIII on MMP2 and MMP9 enzymatic activities, gelatin zymography was performed using cell lysates. The results showed enhanced gelatinolytic activity corresponding to MMP2 and MMP9 in EGFRvIII-overexpressing cells, as indicated by clear degradation bands on the gelatin substrate (Fig. 3C). Conversely, knockdown of EGFRvIII using siEGFRvIII significantly reduced MMP2 and MMP9 mRNA levels, as shown by qPCR analysis (Fig. 3D). This downregulation was corroborated by Western blot analysis, which revealed a decrease in MMP2 and MMP9 protein levels in siEGFRvIII-transfected HSC-2, HSC-3, and SCC-9 cells compared to non-targeting control (N.C.) cells (Fig. 3E). Furthermore, gelatin zymography analysis of siEGFRvIII-transfected cell lysates demonstrated a marked reduction in MMP2 and MMP9 enzymatic activities compared to the N.C. group, further supporting the regulatory role of EGFRvIII in matrix-degrading enzymes (Fig. 3F). These findings indicate that EGFRvIII upregulates both the expression and enzymatic activities of MMP2 and MMP9, enhancing extracellular matrix degradation and promoting invasion. The correlation between EGFRvIII expression and increased MMP2 and MMP9 activity underscores its critical role in modulating matrix remodeling and facilitating OSCC progression.

### EGFRvIII regulates the tumor microenvironment by activating fibroblasts

**Fig. 4:**
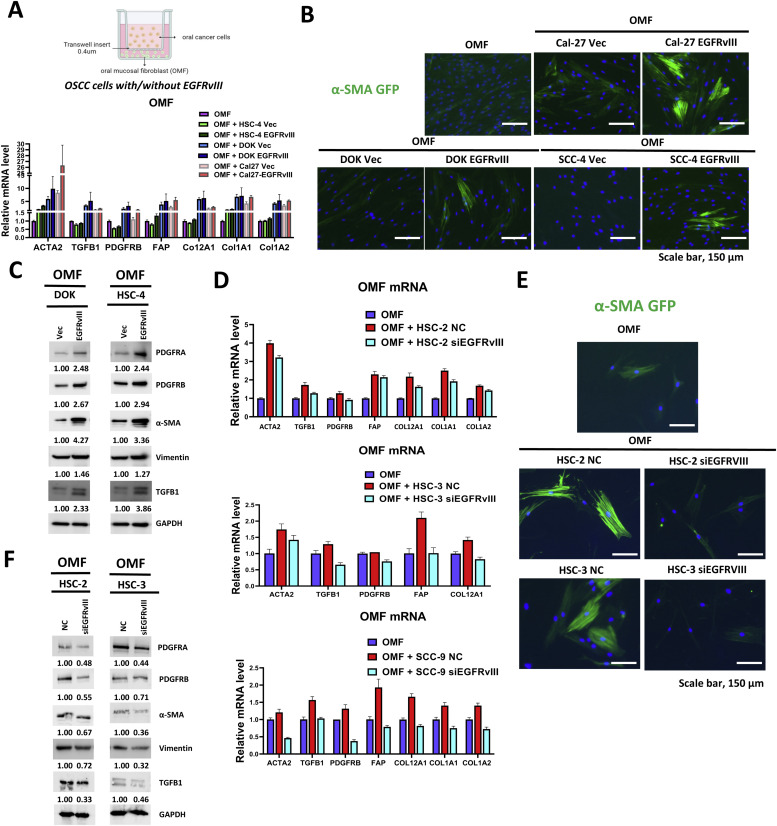
Pre-coculture with EGFRvIII-overexpressing OSCC cells induces fibroblast activation. A. (Top) Schematic representation of the transwell co-culture system used to investigate interactions between OSCC cells and fibroblasts. (Bottom) qPCR analysis of fibroblast activation markers (α-SMA, FAP, TGFβ1, Col12A, Col1A1, Col1A2, and PDGFRβ) in fibroblasts co-cultured with DOK and HSC-4 cells overexpressing EGFRvIII for 72 h. B. Immunofluorescence staining of α-SMA in fibroblasts co-cultured with OSCC cells for 72 h. Scale bar, 150 μm. C. Western blot analysis of fibroblast activation markers (α-SMA, Vimentin, TGFβ1, PDGFRα, and PDGFRβ) in fibroblasts co-cultured with DOK and HSC-4 cells overexpressing EGFRvIII for 72 h. GAPDH was used as a loading control. Protein band intensities were quantified by densitometry, normalized to GAPDH, and presented as numerical values below the gels. d-F. Analyses of fibroblasts co-cultured with EGFRvIII-knockdown OSCC cells (HSC-2 and HSC-3) for 72 h. Knockdown of EGFRvIII in these cells reduced fibroblast activation markers as confirmed by qPCR, immunofluorescence staining, and Western blot analyses.

To further elucidate the role of EGFRvIII in modulating the TME, we conducted co-culture experiments using a transwell system, in which oral mucosal fibroblasts (OMFs) were exposed to EGFRvIII-overexpressing OSCC cell lines, including DOK, Cal-27, and HSC-4 cells. OMFs co-cultured with EGFRvIII-overexpressing cells exhibited significantly elevated expression of fibroblast activation markers and extracellular matrix remodeling genes, including ACTA2 (α-SMA), TGFB1, PDGFRB, FAP, COL1A1, COL1A2, and COL12A1, as measured by RT-qPCR (Fig. 4A). Immunofluorescence analysis confirmed a marked upregulation of α-SMA in OMFs co-cultured with EGFRvIII-overexpressing cells, emphasizing EGFRvIII’s role in promoting myofibroblast differentiation (Fig. 4B). Western blot analysis further validated these findings, showing increased protein levels of α-SMA, Vimentin, TGFβ1, PDGFRA, and PDGFRB in OMFs co-cultured with EGFRvIII-overexpressing cells (Fig. 4C). In contrast, OMFs co-cultured with siEGFRvIII-transfected OSCC cell lines, including HSC-2, HSC-3, and SCC9 cells, showed a significant reduction in the expression of activation markers such as α-SMA, Vimentin, and TGFβ1. This reduction was confirmed through qPCR, Western blot, and immunofluorescence analyses, highlighting a clear difference compared to fibroblasts co-cultured with control cells (Fig. 4D-F). These findings highlight EGFRvIII’s critical role as a central regulator of fibroblast activation, demonstrating its profound impact on stromal remodeling and the induction of myofibroblast differentiation within the TME. Collectively, this evidence underscores EGFRvIII’s dual function in OSCC progression: not only as a potent tumor cell-intrinsic oncogene driving tumor growth and invasion but also as a key orchestrator of pro-tumorigenic microenvironment remodeling.

### EGFRvIII promotes fibroblast recruitment and tumor cell migration

**Fig. 5:**
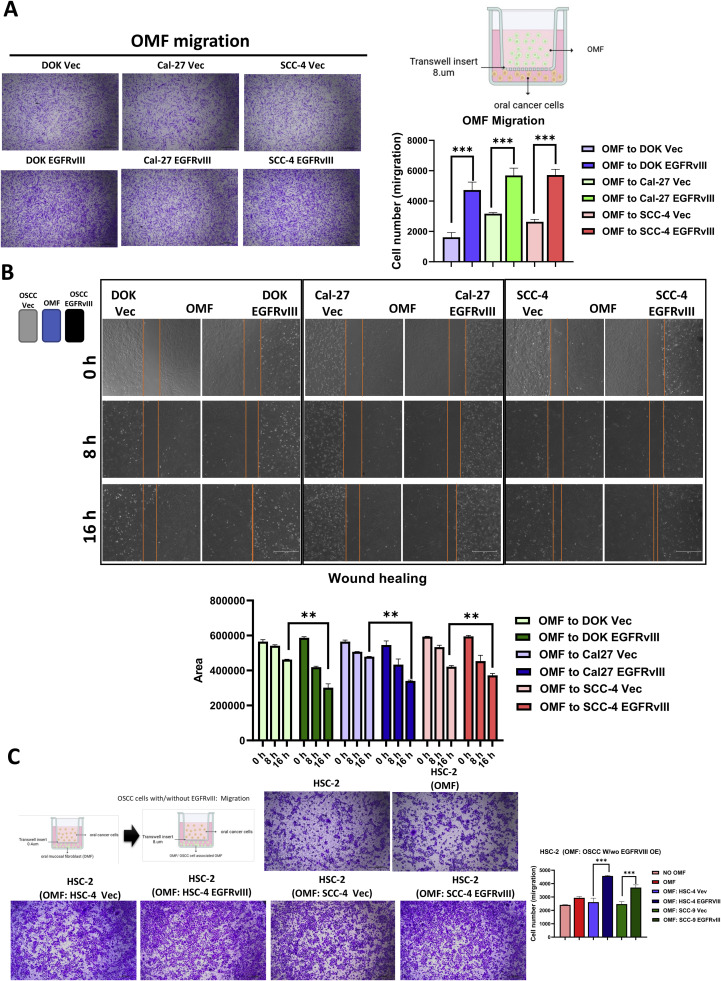
EGFRvIII overexpression in OSCC enhances fibroblast activation and promotes tumor cell migration. A. Migration assays were conducted for 24 h using fibroblasts co-cultured with DOK, Cal-27, and HSC-4 cells transfected with EGFRvIII or control vector for 72 h. B. Wound healing assays of DOK, Cal-27, and HSC-4 cells expressing EGFRvIII or control vector co-cultured with fibroblasts for 72 h. Images were captured at 0, 8, and 16 h. Scale bar, 750 μm.C. Transwell migration assays were performed for 24 h using HSC-2 cells co-cultured with fibroblasts pre-conditioned for 72 h by DOK, Cal-27, and HSC-4 cells expressing EGFRvIII or control vector. Quantitative analysis was performed to assess migration efficiency. Scale bar, 20 μm. Statistical significance was determined using appropriate tests, with *p < 0.05, **p < 0.01, ***p < 0.001.

We hypothesized that EGFRvIII overexpression in OSCC cells not only activates fibroblasts but also recruits them to the tumor microenvironment. To test this hypothesis, we conducted transwell migration and wound-healing assays, exposing OMFs to EGFRvIII-overexpressing OSCC cell lines, including DOK, Cal-27, and HSC-4 cells. The results demonstrated that EGFRvIII-overexpressing OSCC cells attracted fibroblasts more effectively than control cells. Quantitative analysis revealed a significant increase in fibroblast migration toward EGFRvIII-overexpressing tumor cells (Fig. 5A). Similar patterns were observed in wound-healing assays (Fig. 5B), further validating the enhanced recruitment capability of EGFRvIII-overexpressing cells. Interestingly, fibroblasts recruited by EGFRvIII-overexpressing OSCC cells subsequently enhanced tumor cell migration and invasion, establishing a positive feedback loop that amplifies tumor-stroma interactions (Fig. 5C). In contrast, OSCC cells transfected with siEGFRvIII exhibited significantly reduced fibroblast recruitment, as confirmed by migration assays (**Supplemental Fig. 4**). These findings underscore a bidirectional interaction, wherein EGFRvIII-expressing tumor cells actively recruit fibroblasts, which in turn promote tumor cell aggressiveness. This mutual reinforcement between tumor cells and fibroblasts contributes to tumor-stroma synergy and plays a pivotal role in OSCC progression.

### LCN2 induces fibroblast activation via STAT3 signaling

**Fig. 6:**
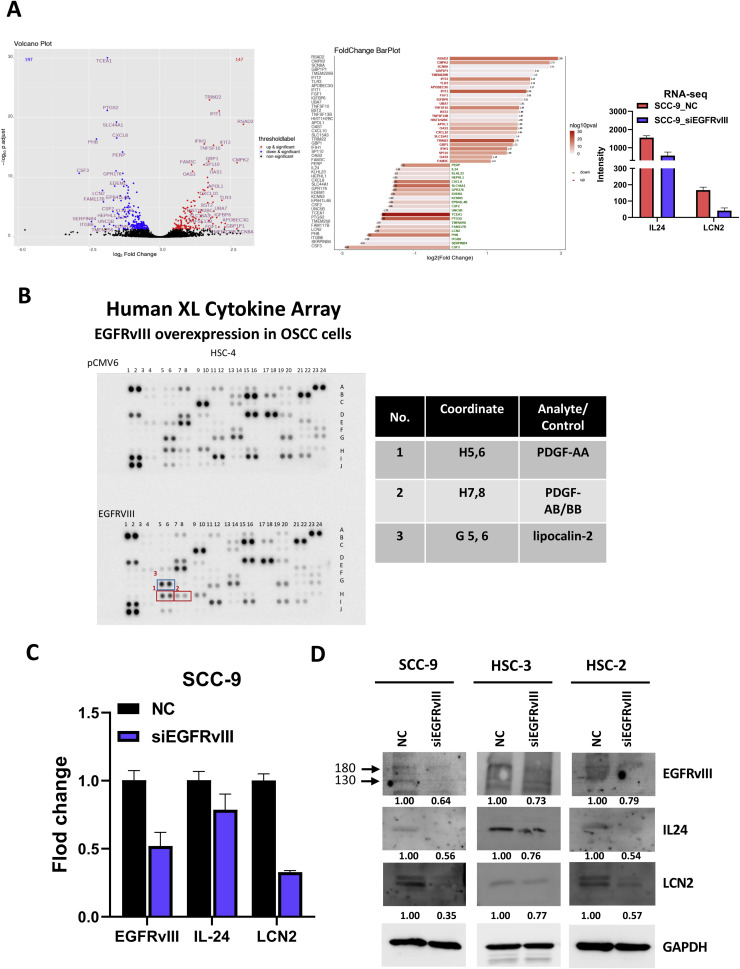
Inhibited expression of IL24 and LCN2 in EGFRvIII knockdown OSCC cells. A. RNA-seq analysis was conducted on SCC-9 cells transfected with siEGFRvIII or non-targeting control (NC) for 72 h. The analysis revealed significant changes in gene expression, including downregulation of cytokines and regulatory proteins such as IL24 and LCN2 in EGFRvIII knockdown cells. B. Proteome analysis was performed using the Proteome Profiler Human XL Cytokine Array Kit. Equal amounts of cell lysates were collected from HSC-4 OSCC cells transfected with either EGFRvIII overexpression vector or control vector for 72 h, followed by selection with a drug for 7 days to establish stable expression cell lines. The cytokine array identified elevated levels of PDGF-AA, PDGF-AB/BB, and LCN2 in EGFRvIII-overexpressing cells compared to controls. C. Quantitative PCR (qPCR) analysis was conducted on SCC-9 cells transfected with siEGFRvIII or NC for 72 h. Results confirmed a significant reduction in the expression levels of EGFRvIII, LCN2, and IL24 in siEGFRvIII-transfected cells compared to NC-transfected cells. GAPDH was used as the reference gene. D. Western blot analysis of EGFRvIII, LCN2, and IL24 protein levels in SCC-9, HSC-2, and HSC-3 cells transfected with siEGFRvIII or NC for 72 h. GAPDH was used as a loading control. Protein band intensities were quantified by densitometry, normalized to GAPDH, and presented as numerical values below the gels. Statistical analyses were performed using appropriate tests, with significance denoted as *p < 0.05, **p < 0.01, ***p < 0.001.

**Fig. 7:**
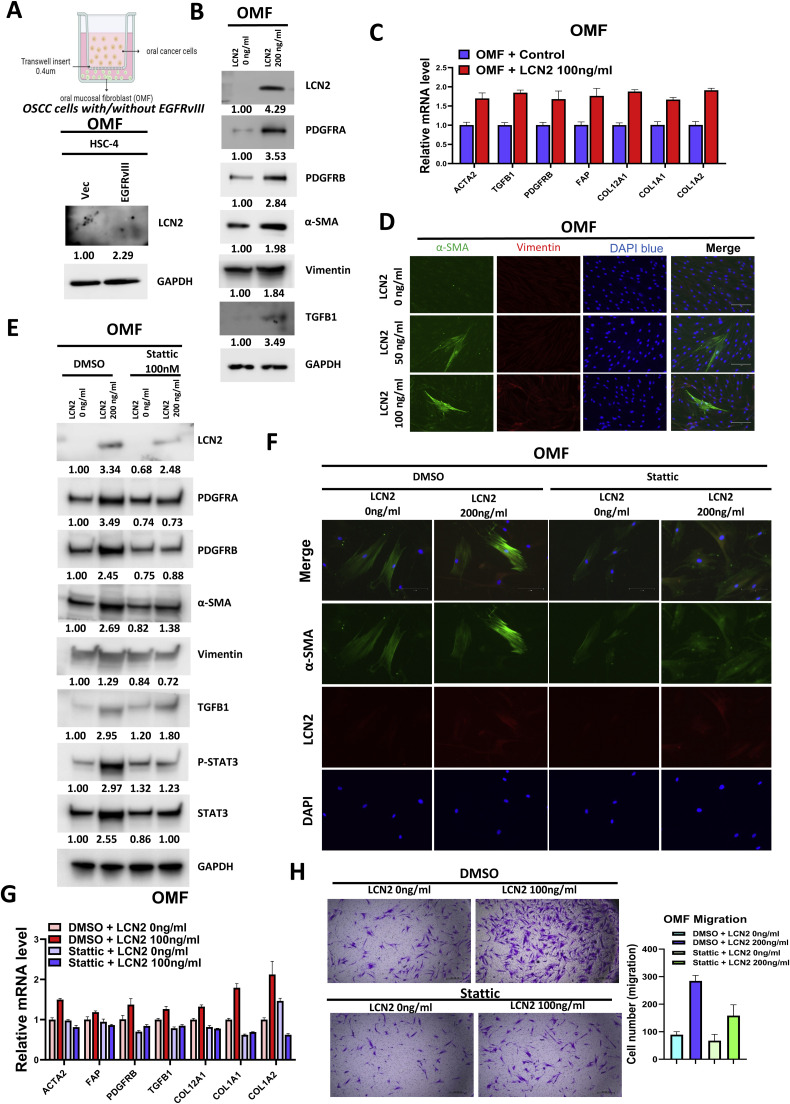
Lipocalin-2 (LCN2) induces fibroblast activation via STAT3 signaling. A. Western blot analysis of LCN2 expression in oral mucosal fibroblasts (OMFs) co-cultured with HSC-4 cells expressing EGFRvIII or control vector for 72 h. GAPDH was used as a loading control. Protein band intensities were quantified by densitometry, normalized to GAPDH, and presented as numerical values below the gels. B. Western blot analysis of fibroblast activation markers (α-SMA, Vimentin, TGFβ1, PDGFRα, and PDGFRβ) in OMFs treated with LCN2 (0 or 100 ng/mL) for 72 h. GAPDH was used as a loading control. Protein band intensities were quantified by densitometry, normalized to GAPDH, and presented as numerical values below the gels. C. qPCR analysis of fibroblast activation markers (α-SMA, FAP, TGFβ1, Col12A, Col1A1, Col1A2, and PDGFRβ) in OMFs treated with LCN2 (0 or 100 ng/mL) for 72 h. GAPDH was used as a reference gene. D. Immunofluorescence staining of fibroblasts treated with LCN2 (0, 50, and 100 ng/mL) for 72 h, showing α-SMA (green), Vimentin (red), and nuclei stained with DAPI (blue). Scale bar, 150 μm. E. Western blot analysis of fibroblast activation markers (α-SMA, Vimentin, TGFβ1, PDGFRα, PDGFRβ, STAT3, and P-STAT3) in OMFs treated with LCN2 (200 ng/mL) in the presence or absence of Stattic (100 nM, a STAT3 inhibitor) for 72 h. Protein band intensities were quantified by densitometry, normalized to GAPDH, and presented as numerical values below the gels. F. Immunofluorescence staining of fibroblasts treated with LCN2 (200 ng/mL) with or without Stattic for 72 h, showing α-SMA (green), LCN2 (red), and nuclei stained with DAPI (blue). Scale bar, 50 μm. G. qPCR analysis of fibroblast activation markers (α-SMA, FAP, TGFβ1, Col12A, Col1A1, Col1A2, and PDGFRβ) in OMFs treated with LCN2 (200 ng/mL) with or without Stattic for 72 h. Scale bar, 150 μm. H. Representative images and quantification of Transwell migration assays performed over 24 h. OMFs were treated with LCN2 (200 ng/mL) with or without Stattic (1 μM). Error bars represent the mean ± standard deviation from three independent experiments. Statistical significance: *p < 0.05, **p < 0.01, ***p < 0.001.

To identify EGFRvIII-regulated genes involved in fibroblast activation, RNA-seq analysis was conducted on RNA isolated from SCC-9 cells transfected with siEGFRvIII or non-targeting control (NC). Differential expression analysis revealed significant downregulation (≥2-fold, *p* < 0.0001) of several mRNAs in the absence of EGFRvIII (Fig. 6A), consistent across at least two OSCC cell lines. Among these, IL24 and LCN2 were notably downregulated upon EGFRvIII knockdown, indicating their positive regulation by EGFRvIII. Cytokine array analysis of lysates from HSC-4 cells overexpressing EGFRvIII (HSC-4-pCMV6-EGFRvIII) or control vector (HSC-4-pCMV6) revealed elevated secretion of several factors, including PDGF-AA, PDGF-AB/BB, and LCN2 in EGFRvIII-overexpressing cells (Fig. 6B). This was further supported by cytokine array results from HSC-2 cells, where LCN2 expression was significantly suppressed in siEGFRvIII-transfected cells compared to controls (**Supplemental Fig. 5A**). Validation through qPCR and Western blot analyses in SCC-9-siEGFRvIII cells confirmed the downregulation of LCN2. Additionally, ELISA demonstrated significantly elevated LCN2 levels in the conditioned medium of EGFRvIII-overexpressing OSCC cells compared to controls (**Supplemental Fig. 5B**). Importantly, using a transwell co-culture system that prevents direct cell–cell contact, we observed that OMFs exposed to EGFRvIII-overexpressing OSCC cells exhibited significantly increased LCN2 levels (Fig. 7A), supporting a paracrine mechanism of LCN2-mediated tumor–stroma communication. To elucidate how LCN2 modulates fibroblast activation, OMFs were treated with recombinant LCN2 (0 or 200 ng/mL) for 72 h or transfected to overexpress LCN2. Both treatments significantly increased the protein levels of α-SMA and vimentin (Fig. 7B, **Supplemental Fig. 6**). RT-qPCR analysis revealed marked upregulation of fibroblast activation markers, including ACTA2 (α-SMA), TGFB1, PDGFRB, FAP, COL1A1, COL1A2, and COL12A1, in response to LCN2 treatment (Fig. 7C). Immunofluorescence analysis corroborated these findings, showing a dose-dependent increase in α-SMA and vimentin expression (Fig. 7D), indicating that LCN2 promotes myofibroblast activation and extracellular matrix remodeling. In contrast, recombinant IL-24 protein induced only a modest increase in α-SMA expression and STAT3 phosphorylation in fibroblasts (Supplemental Fig. 7), suggesting a limited capacity to promote fibroblast activation compared to LCN2. These results indicate that while both IL-24 and LCN2 are transcriptionally regulated by EGFRvIII, only LCN2 exerts a robust functional effect on fibroblast phenotype, and thus plays a dominant mechanistic role in TME remodeling.

To determine whether LCN2 functionally contributes to fibroblast activation, we performed LCN2 knockdown in EGFRvIII-overexpressing OSCC cells (HSC-2 and HSC-3), followed by transwell co-culture with OMFs. LCN2 silencing significantly reduced the expression of fibroblast activation markers, including ACTA2, TGFB1, PDGFRB, and vimentin (**Supplemental Fig. 8A-B**). These findings support the role of LCN2 as a necessary paracrine effector of EGFRvIII-driven stromal remodeling and CAF conversion. LCN2 is known to regulate multiple downstream signaling pathways, including PI3K/AKT, STAT3, ERK/MAPK, NF-κB, and Wnt/β-catenin. Among these, STAT3 has been particularly implicated in fibroblast activation and the maintenance of a cancer-associated phenotype [43,44]. To identify the specific pathways associated with LCN2 overexpression in OMFs, we performed Human Phospho-Kinase Array analysis on lysates from OMF-pCMV6-LCN2 and OMF-pCMV6 control cells. This analysis revealed a significant upregulation of phosphorylated STAT3 (P-STAT3) in LCN2-overexpressing OMFs or OMFs treated with recombinant LCN2 (**Supplemental Fig. 9**). These findings align with previous studies showing that STAT3 phosphorylation stabilizes the activated phenotype of cancer-associated fibroblasts (CAFs) [45,46]. To confirm the role of STAT3 in LCN2-mediated fibroblast activation, Stattic, a STAT3-specific inhibitor, was applied. Stattic treatment effectively suppressed LCN2-induced upregulation of fibroblast activation markers, including α-SMA and vimentin (Fig. 7E, G). Furthermore, Stattic significantly inhibited LCN2-induced fibroblast migration (Fig. 7H), emphasizing the central role of STAT3 in this process. These results establish that LCN2 promotes fibroblast activation and migration via the STAT3 signaling pathway, highlighting a critical mechanism by which EGFRvIII remodels the tumor microenvironment. LCN2 emerges as a pivotal secreted factor that links EGFRvIII expression in tumor cells to fibroblast activation, thereby driving stromal remodeling and contributing to OSCC progression.

### NNK and arecoline enhance EGFRvIII expression in OSCC

**Fig. 8:**
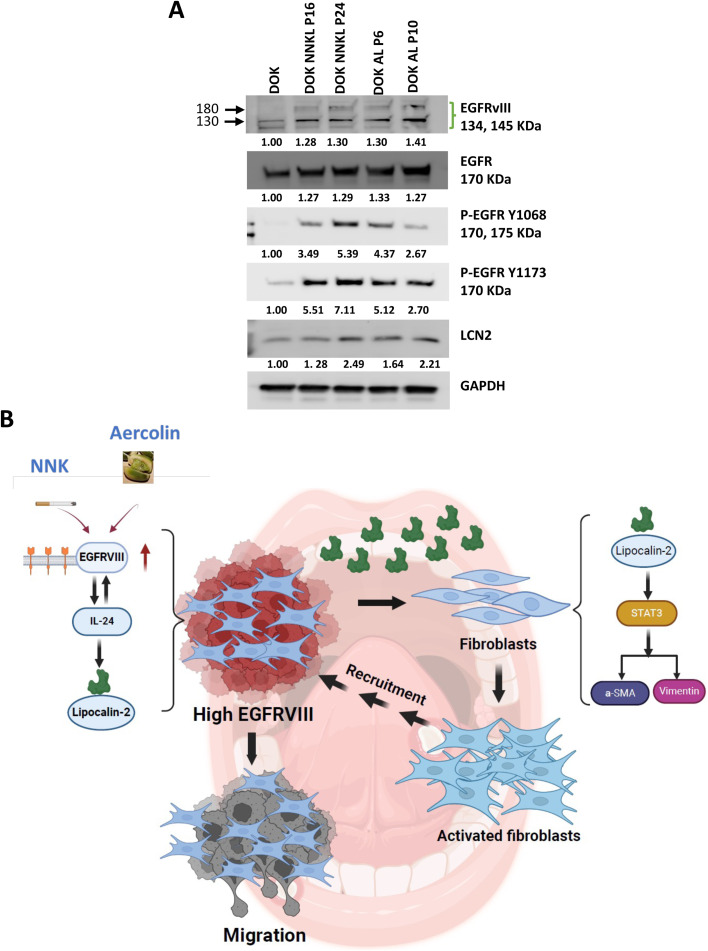
NNK and arecoline-induced OSCC expression of EGFR, EGFRvIII, and LCN2. A. Western blot analysis of EGFR, EGFRvIII, and LCN2 protein levels in DOK cells treated with non-toxic concentrations of NNK (10 μM) and arecoline (50 μM) for 12 months. The carcinogen-transformed cells were designated as NNK-L (NNK-tolerant) and Are-L (arecoline-tolerant). GAPDH was used as a loading control. B. Schematic diagram illustrating the role of EGFRvIII in OSCC cells. EGFRvIII induces LCN2 secretion, which promotes fibroblast activation and recruitment. These activated fibroblasts further enhance tumor cell migration, demonstrating a synergistic interaction between tumor cells and the tumor microenvironment.

Environmental carcinogens such as NNK and arecoline have been shown to amplify EGFR related signaling pathways [47]. Long-term exposure of DOK cells to non-toxic concentrations of NNK (10 μM) and arecoline (50 μM) for 12 months resulted in significantly elevated expression levels of EGFR, EGFRvIII, and, LCN2, as confirmed by Western blot analysis. Additionally, phosphorylation of EGFR at Y1173 and Y1068 was enhanced, signifying increased EGFR signaling activity (Fig. 8A). These results underscore the role of environmental carcinogens in amplifying EGFRvIII-driven oncogenic pathways, linking external factors to OSCC progression.

### The IL-24/EGFRvIII/STAT3 loop in OSCC cells

To investigate the interplay between IL-24, EGFRvIII, and STAT3 signaling in OSCC, Western blot analyses were performed on cells with EGFRvIII overexpression or knockdown. In HSC-4 cells transfected with the EGFRvIII overexpression vector (OE), protein levels of EGFRvIII, IL-24, and LCN2 were markedly elevated compared to cells transfected with the control vector (Vec). Conversely, knockdown of EGFRvIII in SCC-9 cells using siEGFRvIII significantly reduced the expression of these factors compared to non-targeting control (NC) cells (**Supplemental Fig. 10A**). We further explored whether IL-24 modulates EGFRvIII and its associated downstream signaling pathways. In HSC-4 cells transfected with an IL-24 overexpression vector (OE), Western blot analysis revealed increased levels of EGFRvIII, total EGFR, phosphorylated EGFR (Y1173 and Y1068), LCN2, total STAT3, and phosphorylated STAT3 (Y705) compared to cells transfected with the control vector (Vec) (**Supplemental Fig. 10B**). These findings demonstrate that IL-24 overexpression enhances EGFRvIII expression and activates both the EGFR and STAT3 signaling axes, contributing to the upregulation of tumor-promoting factors. Together, these results establish a positive feedback loop, wherein IL-24 amplifies EGFRvIII expression and its downstream signaling pathways, reinforcing oncogenic processes in OSCC cells. These results establish a direct connection between environmental carcinogens and the upregulation of EGFRvIII and its downstream effectors, emphasizing their real-world relevance to OSCC progression. Collectively, our data demonstrate that EGFRvIII expression in OSCC cells not only drives intrinsic tumor growth, migration, and invasion, but also reshapes the tumor microenvironment (TME). Through the induction of LCN2, EGFRvIII-expressing tumor cells actively recruit and activate fibroblasts, which subsequently enhance tumor cell aggressiveness. This bidirectional interaction creates a positive feedback loop between tumor cells and fibroblasts (Fig. 8B).

## Discussion

This study highlights EGFRvIII as a key driver of OSCC progression and tumor microenvironment (TME) remodeling, particularly through cancer-associated fibroblast (CAF) activation and recruitment. Our findings establish EGFRvIII as a potent oncogenic factor in OSCC and a major regulator of tumor-stroma interactions, providing a foundation for targeted therapeutic strategies. EGFRvIII is a constitutively active, ligand-independent mutant of EGFR, best characterized in glioblastoma, where it promotes tumor aggressiveness, intratumoral heterogeneity, and resistance to conventional therapies. Accumulating preclinical evidence indicates that EGFRvIII-expressing glioblastoma cells enhance proliferation, invasion, angiogenesis, stemness, and therapy resistance across multiple model systems [17,[48], [49], [50]]. In this study, we demonstrate that EGFRvIII is highly prevalent in OSCC, with approximately 70 % of tumor specimens showing expression as determined by RT-PCR targeting the exon 1–8 junction, a unique sequence generated by the exon 2–7 deletion. The molecular identity of this product was further validated by Sanger sequencing. While the present study focuses on transcript-level analysis, protein-level expression of EGFRvIII was previously confirmed in our published work, using immunohistochemistry with an EGFRvIII-specific antibody [18]. In that earlier study involving 108 clinical OSCC cases, 75.0 % of tumor samples exhibited moderate to strong EGFRvIII staining, with 31.5 % classified as having high expression levels. EGFRvIII protein expression was significantly associated with advanced tumor stage, increased tumor size, and poor overall survival. In addition, high EGFRvIII expression showed a significant correlation with advanced clinical stage and higher tumor (T) classification, further supporting its role as a marker of aggressive disease phenotype [18]. Together, these results underscore EGFRvIII’s dual functional and prognostic roles in OSCC, supporting its potential as both a biomarker for risk stratification and a target for precision therapy. Mechanistically, EGFRvIII drives oncogenic signaling by constitutively activating PI3K/AKT, Ras/MAPK, and JAK/STAT pathways, which promote OSCC cell proliferation, survival, and migration [[50], [51], [52]]. The altered extracellular domain confers ligand independence, amplifying oncogenic signaling and enhancing angiogenesis and epithelial–mesenchymal transition (EMT)—hallmarks of aggressive tumors and therapeutic resistance [[53], [54], [55]]. Consistently, our results show that EGFRvIII overexpression significantly enhances OSCC cell proliferation, migration, and invasion, while EGFRvIII knockdown reverses these effects (Fig. 2). Moreover, EGFRvIII modulates the TME by altering cytokine and growth factor secretion, influencing stromal fibroblast activity, immune infiltration, and ECM remodeling [[55], [56], [57], [58]]. A crucial mechanism by which EGFRvIII enhances OSCC invasion is through upregulation of matrix metalloproteinases (MMPs), particularly MMP2 and MMP9, which degrade ECM components to facilitate tumor dissemination [[59], [60], [61]]. Studies in glioblastoma have demonstrated that EGFRvIII expression directly regulates the release of MMP2 and MMP9, leading to ECM breakdown and enhanced tumor invasiveness [52,62]. We observed that EGFRvIII-overexpressing OSCC cells significantly increase MMP2 and MMP9 mRNA and protein levels, reinforcing its role in ECM remodeling and metastasis. Given the oral cavity’s chronic exposure to carcinogens (e.g., tobacco, alcohol, areca nut) and microorganisms, persistent inflammation triggers fibroblast activation [63]. Fibroblasts, in turn, enhance tumor progression through cytokine secretion, ECM remodeling, and pro-invasive signaling [64,65]. EGFRvIII likely induces CAF formation by modulating fibroblast-activating cytokines. IL-8, for example, has been shown to activate fibroblasts via STAT3 phosphorylation, upregulating MMP1 and promoting tumor cell invasion [66]. Consistently, our co-culture experiments reveal that EGFRvIII-expressing OSCC cells induce fibroblast activation, marked by elevated α-SMA and vimentin expression—hallmarks of CAF transformation (Fig. 5). Moreover, EGFRvIII-induced CAFs further enhance OSCC migration, forming a positive feedback loop similar to CAF-driven invasion in breast cancer [31,[67], [68], [69]].

We identify Lipocalin-2 (LCN2) as a critical mediator linking EGFRvIII to TME remodeling in OSCC. Using ELISA, cytokine arrays, and RNA-seq, we demonstrate that LCN2 secretion is upregulated in EGFRvIII-expressing OSCC cells, suggesting its role in fibroblast activation and ECM modulation (Fig. 7). LCN2, originally found in neutrophils and kidney cells, is implicated in tumor invasion, immune suppression, and ECM degradation in various cancers, including thyroid, prostate, lung, liver, and OSCC [[70], [71], [72], [73], [74], [75], [76]]. Elevated LCN2 levels have been observed in fibroblasts, endothelial cells, and tumor-associated macrophages (TAMs), reinforcing its role in TME regulation [77,78]. LCN2 also promotes VEGF-mediated lymphangiogenesis [79] and tumor metastasis via epithelial–mesenchymal transition (EMT) [80,81]. Beyond cancer, LCN2 is associated with fibrosis, where it correlates with COL1A1 expression and drives ECM remodeling in liver fibrosis [82]. Our data further demonstrate that tumor-derived LCN2 plays a critical role in mediating EGFRvIII-induced fibroblast activation via the STAT3 signaling pathway. Co-culture of OMFs with EGFRvIII-overexpressing OSCC cells led to marked upregulation of fibroblast activation markers, including α-SMA, PDGFRA, PDGFRB, and vimentin (Fig. 4). However, silencing LCN2 in tumor cells significantly attenuated these markers and reduced STAT3 phosphorylation in fibroblasts, indicating that LCN2 is a necessary paracrine effector downstream of EGFRvIII **(Supplemental Fig. 8)**. These findings fulfill the criteria for causal inference and highlight LCN2 as a viable therapeutic target for disrupting tumor–stroma crosstalk in OSCC. Beyond its role in fibroblast activation, LCN2 regulates several oncogenic signaling pathways—including PI3K/AKT, STAT3, ERK/MAPK, NF-κB, and Wnt/β-catenin—that collectively promote tumor proliferation, immune evasion, and extracellular matrix remodeling [43,44]. Notably, EGFRvIII-induced LCN2 secretion establishes a bidirectional signaling loop with fibroblasts. LCN2 binds to SLC22A17 receptors on fibroblasts, activating STAT3, which further enhances fibroblast proliferation and secretion of MMPs and IL-6, reinforcing tumor growth and inflammation [83,84]. Our results suggest that targeting the EGFRvIII–LCN2–STAT3 axis could disrupt this oncogenic feedback loop and impair tumor progression. mRNA vaccines targeting EGFRvIII are emerging as a promising immunotherapy. Unlike traditional approaches, these vaccines use lipid nanoparticle-encapsulated mRNA encoding the EGFRvIII extracellular domain, effectively stimulating cytotoxic T lymphocytes (CTLs) and triggering anti-tumor immune responses [85]. This strategy could be especially beneficial for OSCC patients with high EGFRvIII expression, selectively eliminating tumor cells while sparing normal tissues. We further explored the impact of environmental carcinogens (NNK and arecoline) on EGFRvIII expression. Prolonged exposure to these agents upregulates EGFRvIII, LCN2, and other oncogenic factors, while also enhancing EGFR phosphorylation at Y1173/Y1068—key sites for downstream activation. Given the high OSCC incidence in regions with prevalent areca nut and tobacco use, these findings highlight the importance of environmental regulation of EGFRvIII pathways. Targeting EGFRvIII alone may be insufficient, given its interplay with LCN2, STAT3, and CAFs. Therefore, combination therapies integrating anti-stromal agents (LCN2/STAT3 inhibitors) with immunomodulatory strategies could enhance treatment efficacy. Future studies should explore: Development of EGFRvIII-targeting therapies, including mRNA vaccines and small-molecule inhibitors for LCN2/STAT3. The role of additional environmental factors in modulating EGFRvIII expression and identifying biomarkers for high-risk OSCC patients. Combination therapies that integrate anti-stromal and immunomodulatory approaches to improve clinical outcomes.

This study identifies EGFRvIII as a central driver of OSCC progression and TME remodeling, with LCN2 emerging as a key downstream effector. By promoting CAF activation, ECM remodeling, and STAT3-mediated signaling, LCN2 establishes a crucial link between EGFRvIII-expressing tumor cells and stromal reprogramming, ultimately enhancing tumor growth and invasion. Targeting the EGFRvIII–LCN2–STAT3 axis represents a promising therapeutic strategy for OSCC. Future research should focus on developing EGFRvIII-targeting approaches, including mRNA vaccines and LCN2/STAT3 inhibitors, while exploring combination therapies that simultaneously address tumor-intrinsic and TME-mediated mechanisms. Disrupting this oncogenic axis holds potential for improving clinical outcomes and providing novel treatment options for EGFRvIII-expressing OSCC patients.

## Data accessibility

All data supporting the findings of this study are available within the paper and its Supplementary Information. Additional data can be provided upon reasonable request.

## Ethical approval

All human oral tissue samples were obtained from patients at the Taipei Medical University Hospital (TUH) with the understanding and written consent of each subject. The study was approved by the Taipei Medical University Hospital (TUH) Institutional Review Board (approval number: N202204007). The study methodologies conformed to the standards set by the Declaration of Helsinki.

All animal experiments complied with institutional guidelines and were approved by the Institutional Animal Care and Utilization Committee (IACUC) of Taipei Medical University, Taipei, Taiwan.

## Methods

RNA Extraction and Quantitative Real-Time PCR (qRT-PCR)

Protein Extraction and Western Blot Analysis

CCK-8 Assay

Colony Formation Assay

Zymographic Analysis

Enzyme-Linked Immunosorbent Assay (ELISA)

Hematoxylin and Eosin (H&E) Staining

## Funding

This study was supported by the Research Center of Cancer Translational Medicine at Taipei Medical University, sponsored by the Higher Education Sprout Project, Ministry of Education, Taiwan. Additional support was provided by the National Science and Technology Council, Taiwan (NSTC 113-2314-B-038-143), and the Joint Research Program between National Taiwan University Hospital and Taipei Medical University.

## CRediT authorship contribution statement

**Hsuan-Yu Peng:** Writing – original draft, Visualization, Validation, Methodology, Formal analysis, Data curation. **Kwang-Yu Chang:** Visualization, Validation, Formal analysis, Data curation. **Wei-Min Chang:** Validation, Methodology. **Chia-Yu Wu:** Data curation. **Hsin-Lun Lee:** Data curation. **Yung-Chieh Chang:** Methodology, Formal analysis, Data curation. **Ko-Jiunn Liu:** Methodology, Formal analysis, Data curation. **Shine-Gwo Shiah:** Methodology, Formal analysis, Data curation. **Ching-Chuan Kuo:** Formal analysis, Data curation. **Jang-Yang Chang:** Writing – review & editing, Supervision, Resources, Funding acquisition, Conceptualization.

## Declaration of competing interest

The authors declare that they have no known competing financial interests or personal relationships that could have appeared to influence the work reported in this paper.

## Appendix Supplementary materials

Image, application 1
