# Supplementary material for: EGFRvIII-driven microenvironmental fibroblast activation and transformation accelerate oral cancer progression via lipocalin-2/STAT3 axis
Source: Neoplasia. 2025 Jun 4;66:101193. doi: 10.1016/j.neo.2025.101193 (PMC12171556; doi:10.1016/j.neo.2025.101193)
Supplement: Supplementary file 1 [file mmc1.docx]

**Supplementary Materials for**

**EGFRvIII-Driven Microenvironmental Fibroblast Activation and Transformation Accelerate Oral Cancer Progression via Lipocalin-2/STAT3 axis**

**Authors:**

**Hsuan-Yu Peng, Kwang-Yu Chang, Wei-Min Chang, Chia-Yu Wu, Hsin-Lun Lee, Yung-Chieh Chang, Ko-Jiunn Liu, Shine-Gwo Shiah, Ching-Chuan Kuo, Jang-Yang Chang**

***Correspondence:** Jang-Yang Chang,

E‐mail: jychang@tmu.edu.tw, [jychang@nhri.edu.tw](mailto:jychang@nhri.edu.tw)

TMU Research Center of Cancer Translational Medicine, Taipei Medical University Hospital, College of Medicine, Taipei Medical University, No. 252, Wuxing St., Xinyi Dist., Taipei, 110301 Taiwan (R.O.C.)

Institute of Biotechnology and Pharmaceutical Research, National Health Research Institutes, No.35,Keyan Road, Zhunan City, Miaoli 35053,Taiwan (R.O.C.)

**This file includes:**

**Methods**

**Supplementary Figures 1 to 10**

**Methods**

**RNA Extraction and Quantitative Real-Time PCR (qRT-PCR)**

Total RNA was extracted from cell lines using the TRIzol reagent (Life Technologies, Gaithersburg, MD, USA) according to the manufacturer’s protocol. First-strand cDNA was synthesized from 1 µg of total RNA using the Maxima First Strand cDNA Synthesis Kit for qRT-PCR (Thermo Fisher Scientific, Carlsbad, CA, USA). PCR reactions were performed on an Eppendorf Mastercycler Nexus Thermal Cycler (Eppendorf AG, Hamburg, Germany). The expression of target genes, including MMP2, MMP9, EGFRvIII, ACTA2, PDGFRB, FAP, Col1a1, Col1a2, Col12a1, and LCN2, was detected using qRT-PCR with GAPDH as the internal reference gene. The specific primer sequences used for the amplification are as follows:

| Gene Name | Forward Primer (5'-3') | | Reverse Primer (5'-3') |  |
| --- | --- | --- | --- | --- |
| TGFB1 | GGCCAGATCCTGTCCAAGC | GTGGGTTTCCACCATTAGCAC | | |
| Col12a1 | CAAAGGAGGCAATACTCTCACAG | GAAGGTGCTTCAACATCGTCT | |  |
| PDGFRB | AGACACGGGAGAATACTTTTGC | AGTTCCTCGGCATCATTAGGG | |  |
| ACTA2 | GAGCGTGGC TAT TCC TTC GT | TTCAAAGTCCAGAGCTACATAACACAGT | |  |
| FAP | ATC TAT GAC CTT AGC AAT GGA GAA TTT GT | GTT TTG ATA GAC ATA TGC TAA TTT ACT CCC AAC | |  |
| Col1a1 | CTG CTG GAC GTC CTG GTG AA | ACG CTG TCC AGC AAT ACC TTG AG | |  |
| Col1a2 | GAG GGC AAC AGC AGG TTC ACT TA | TCA GCA CCA CCG ATG TCC AA | |  |
| MMP9 | GCC ACT ACT GTG CCT TTG AGT C | CCC TCA GAG AAT CGC CAG TAC T | |  |
| MMP2 | AGC GAG TGG ATG CCG CCT TTA A | CAT TCC AGG CAT CTG CGA TGA G | |  |
| IL24 | CTTCTCTGGAGCCAGGTATCAG | GGCACTCGTGATGTTATCCTGAG | |  |
| NGAL (LCN2) | GTG AGC ACC AAC TAC AAC CAG C | GTTCCGAAGTCAGCTCCTTGGT | |  |
| EGFRvIII | CTC TGG AGG AAA AGA AAG GTA A | AGG CCC TTC GCA CTT CTT AC | |  |
| GAPDH | GTCTCCTCTGACTTCAACAGCG | ACCACCCTGTTGCTGTAGCCAA | |  |

qRT-PCR reactions were prepared using the Maestro Green EvaGreen qPCR Master Mix (Maestrogen, Hsinchu, Xiangshan, Taiwan R.O.C.), following the manufacturer’s instructions. Reactions were run on an Applied Biosystems QuantStudio 3 real-time PCR system. The expression levels of the target genes were quantified based on the threshold cycle (Ct) values. Relative expression levels were calculated using the 2⁻▵▵Ct method, after normalization with the internal reference gene GAPDH. All experiments were performed in triplicate to ensure reproducibility

**Protein Extraction and Western Blot Analysis**

Cells were lysed in RIPA lysis buffer containing 50 mM Tris-HCl, 1% NP-40, 150 mM NaCl, 0.1% SDS, 1 mM PMSF, and 1 mM Na₃VO₄, supplemented with a complete protease and phosphatase inhibitor cocktail (1:1000; Sigma-Aldrich, Inc.). Protein concentrations were quantified using the BCA assay kit (Thermo Scientific, USA) according to the manufacturer’s instructions. Protein lysates (10–30 μg) were separated by SDS-PAGE on 10% polyacrylamide gels and subsequently transferred onto polyvinylidene fluoride (PVDF) membranes (Pall Life Sciences, Glen Cove, NY, USA).

The membranes were blocked and incubated overnight at 4°C with primary antibodies targeting EGFRvIII (31-1305-00, RevMAb Biosciences, USA), phospho-STAT3 (#9145, Cell Signaling Technology, USA), STAT3 (#610189, BD Biosciences, NJ, USA), α-SMA (ab7817, Abcam, Cambridge, UK), TGFβ1 (ab215715, Abcam, Cambridge, UK), PDGFRβ (ab69506, Abcam, Cambridge, UK), Vimentin (IR45-137, iReal Biotechnology, Inc.), MMP2 (IR67-228, iReal Biotechnology, Inc.), MMP9 (#3852, Cell Signaling Technology, USA), NGAL (#26991-1-AP, Proteintech, China), IL-24 (#26772-1-AP, Proteintech, China), α-tubulin (IRM001, iReal Biotechnology, Inc.), and GAPDH (GTX100118, GeneTex, Irvine, CA, USA). GAPDH or α-tubulin were used as internal loading controls.

Following primary antibody incubation, the membranes were washed and incubated with horseradish peroxidase (HRP)-conjugated secondary antibodies at room temperature for 1 hour. Protein bands were detected using enhanced chemiluminescence reagents (PerkinElmer, Waltham, MA, USA) and visualized using the BioSpectrum 810 Imaging System (UVP, Upland, CA, USA). Densitometric analysis of protein bands was performed using ImageJ software (Bethesda, MD, USA). Band intensities were normalized to the corresponding internal control (GAPDH or α-tubulin) for accurate quantification.

**CCK-8 Assay**

OSCC cells were infected with EGFRvIII or a control vector and cultured for 72 hours. To establish stable expression cell lines, cells were treated with a selection drug for an additional 7 days. After selection, equal numbers of cells were plated into 96-well plates at the same density and allowed to grow for 72 hours. For knockdown experiments, OSCC cells were transfected with siEGFRvIII or non-targeting control (N.C.) for 72 hours. Equal numbers of transfected cells were then plated into 96-well plates at the same density and cultured for an additional 72 hours. Cell growth was assessed using the Cell Counting Kit-8 (CCK-8; Sigma-Aldrich, St Louis, USA). Briefly, 10 µL of CCK-8 solution was added to each well, followed by incubation at 37°C for 2 hours. The absorbance at 450 nm was measured using a SpectraMax 250 microplate reader (Molecular Devices, CA, USA).

**Colony Formation Assay**

For the colony formation assay, DOK, Cal-27, HSC-2, HSC-3, HSC-4 cells (150 cells/well) and SCC-4, SCC-9 cells (300 cells/well) were seeded into 6-well plates and cultured for 10 days at 37°C in a humidified incubator with 5% CO₂. After the incubation period, colonies were fixed with 100% ethanol, stained with 1% crystal violet, and counted under a microscope.

**Zymographic Analysis**

Gelatinolytic activities of MMPs were assessed under non-reducing conditions using modified sodium dodecyl sulfate polyacrylamide gel electrophoresis (SDS-PAGE). The analysis was performed on a 7.5% polyacrylamide gel copolymerized with 0.1% gelatin type I. Samples (20 µL) were mixed with 10 µL of loading buffer and loaded onto the gel without prior boiling. Electrophoresis was conducted under non-reducing conditions. Following electrophoresis, gels were washed twice with 2.5% Triton X-100 to remove SDS and allow proteins to renature. The gels were then incubated in renaturation buffer containing 50 mmol/L Tris (pH 7.5), 5 mmol/L CaCl₂, 1 µmol/L ZnCl₂, and 0.01% NaN₃ at 37°C for 18 hours to activate gelatinolytic enzymes. After incubation, gels were stained with 0.2% Coomassie Blue solution (0.25% brilliant blue, 40% methanol, 7.5% acetic acid) for 4 hours at room temperature. Destaining was performed using a buffer containing 30% methanol and 10% acetic acid until clear contrast was achieved. Proteolytic activity was visualized as clear white bands against a blue background of undigested gelatin, indicating areas of gelatin degradation. The gels were documented using the Bio-Rad ChemiDoc MP imaging system (Bio-Rad, Hercules, California, USA) to ensure accurate recording and analysis of gelatinolytic activity.

**Enzyme-Linked Immunosorbent Assay (ELISA)**

Cell supernatants were collected and analyzed for the detection of human Lipocalin-2/NGAL secretion using the R&D Duoset ELISA Kit (Cat# DY1757-05, R&D Systems, Inc., Minneapolis, MN, USA), following the manufacturer’s instructions.

**Hematoxylin and Eosin (H&E) Staining**

Tissue samples were fixed in 10% neutral buffered formalin for 24 hours, embedded in paraffin, and sectioned at 4 µm thickness. The sections were deparaffinized in xylene, rehydrated through a graded ethanol series, and rinsed in distilled water. Hematoxylin staining was performed for 5 minutes to label nuclei, followed by differentiation in 0.3% acid alcohol and a subsequent rinse in tap water. Eosin staining was performed for 2 minutes to counterstain the cytoplasm and extracellular matrix. After dehydration through graded ethanol, the sections were cleared in xylene and mounted with coverslips using a resinous medium. The slides were were scanned at 40× magnification using Aperio Digital Pathology Slide Scanners; high‐power images (40× magnification) were randomly selected and analyzed by leica aperio imagescope digital slide viewer v9.1.19.1568.

**Supplementary Figure**

**Supplemental Figure 1**

**
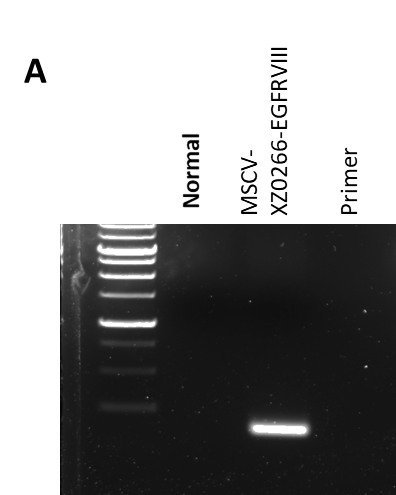
**

**Supplemental Figure 1. Identification of EGFRvIII in human normal oral gingiva at the RNA level**

**A.** Detection of wild-type (WT) EGFR (929 bp) and EGFRvIII (128 bp) in normal oral gingiva by RT-PCR. Lane 2 serves as an EGFRvIII-positive control, while lane 3 contains H₂O to rule out contamination.

**Supplemental Figure 2**

**
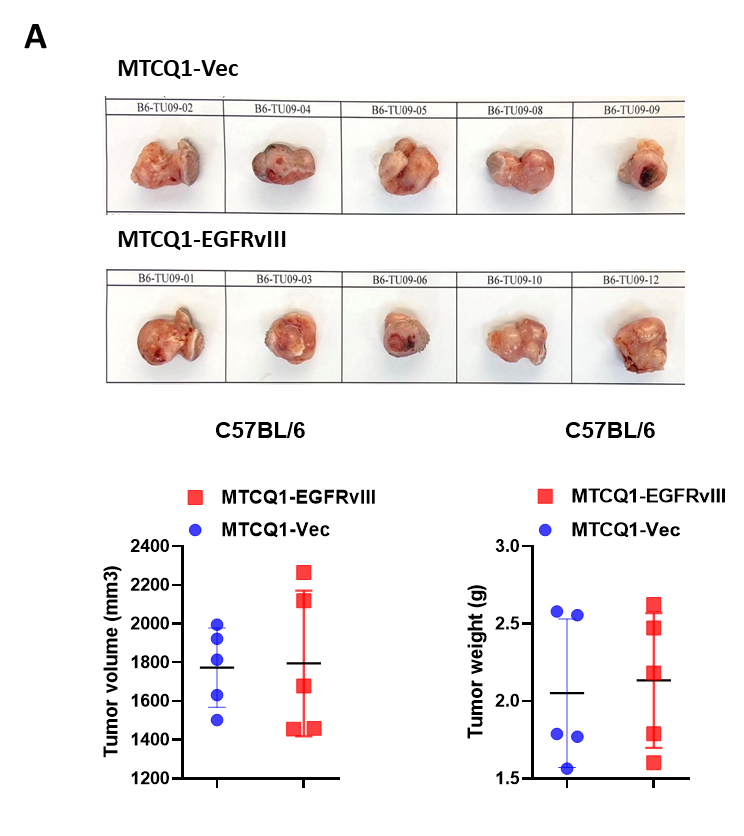
**

**Supplemental Figure 2.** **EGFRvIII overexpression does not affect tumor growth in an OSCC xenograft model**

**A.** Representative images of xenograft tumors on day 35 following subcutaneous injection of MTCQ1 cells (mouse OSCC cells) overexpressing EGFRvIII or a control vector into C57BL/6 mice. Tumors were harvested and analyzed for weight and volume on day 35.

**Supplemental Figure 3**

**
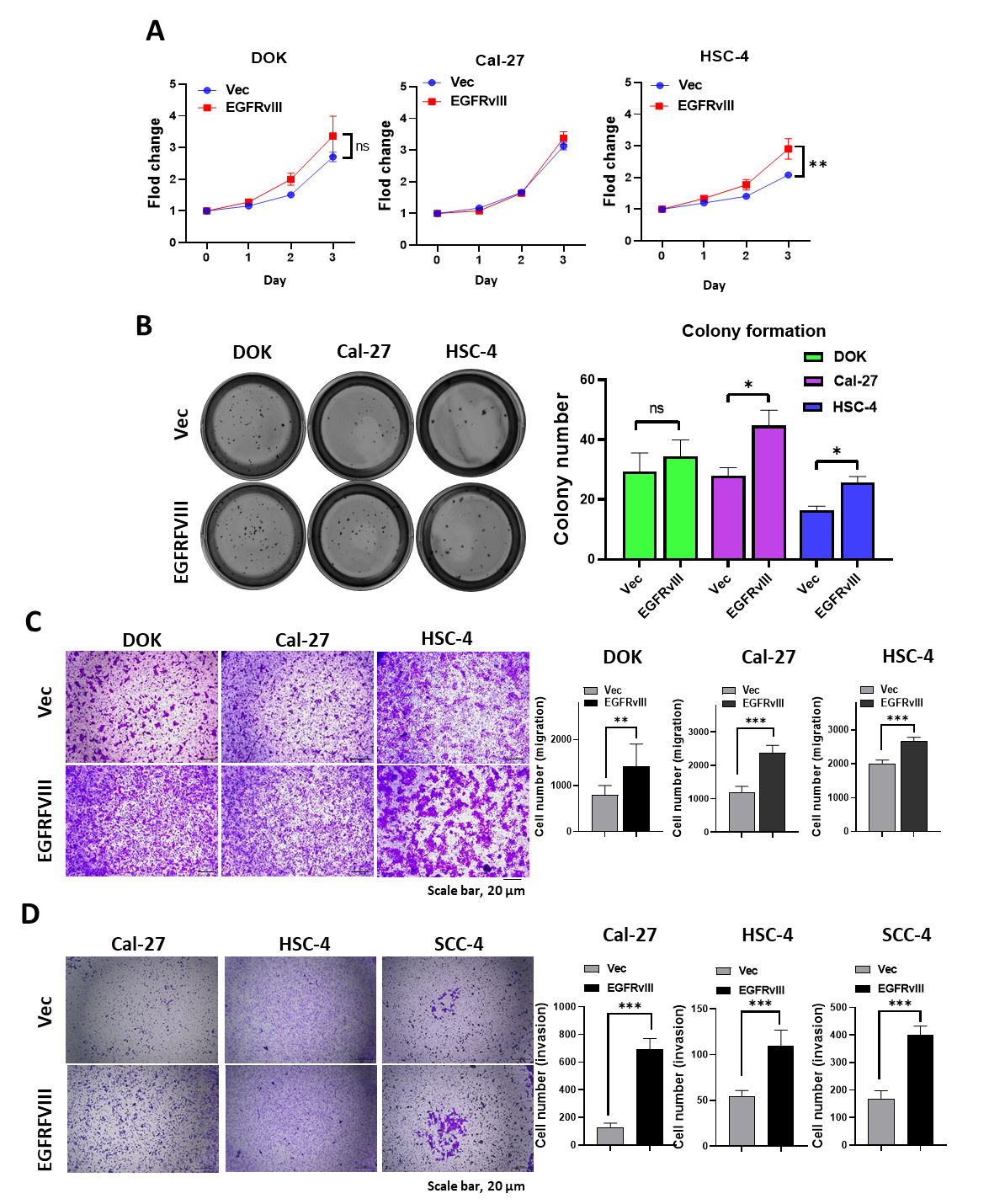
**

**Supplemental Figure 3. EGFRvIII expression enhances OSCC growth, migration, and invasion**

A. Growth curves of DOK, Cal-27, and HSC-4 cells transfected with EGFRvIII or control vector, measured at 24, 48, and 72 hours using CCK-8 assays. Cells were treated with a selection drug for 7 days post-transfection to establish stable expression cell lines, followed by CCK-8 analysis. B. Colony formation assays of DOK, Cal-27, and HSC-4 cells transfected with EGFRvIII or control vector for 72 hours, followed by selection for stable expression over 7 days. Cells were seeded into 6-well plates and cultured for an additional 7 days before colony counting. C. Transwell migration assays were performed for 24 hours using DOK, Cal-27, and HSC-4 cells transfected with EGFRvIII or control vector for 72 hours, followed by selection for stable expression over 7 days. Scale bar, 20 μm. D. Transwell invasion assays using Matrigel-coated inserts were conducted for 24 hours under the same conditions as in (C). Scale bar, 20 μm. Error bars represent the mean ± standard deviation from three independent experiments. Statistical significance was determined using appropriate tests, with significance denoted as *p < 0.05, **p < 0.01, ***p < 0.001.

**Supplemental Figure 4**

**
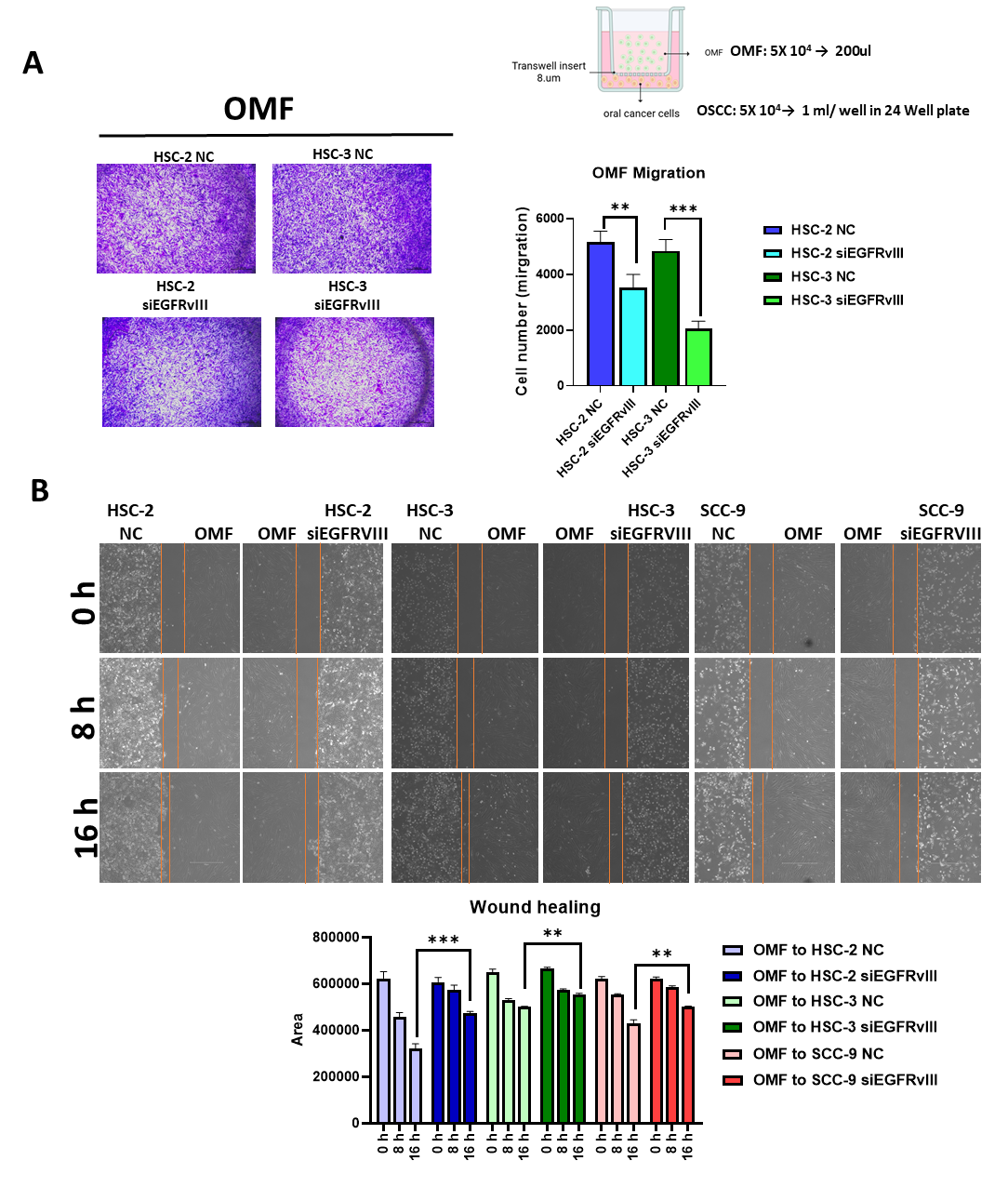
**

**Supplemental Figure 4. EGFRvIII knockdown in oral cancer cells reduces fibroblast recruitment**

**A.** Migration assays were conducted for 24 hours using fibroblasts co-cultured with SCC-9, HSC-2, and HSC-3 cells transfected with siEGFRvIII or NC for 72 hours. **B.** Wound healing assays of SCC-9, HSC-2, and HSC-3 cells transfected with siEGFRvIII or NC for 72 hours and co-cultured with fibroblasts. Images were captured at 0, 8, and 16 hours. Scale bar, 750 μm. Statistical significance was determined using appropriate tests (*p < 0.05, **p < 0.01, ***p < 0.001).

**Supplemental Figure 5**

**
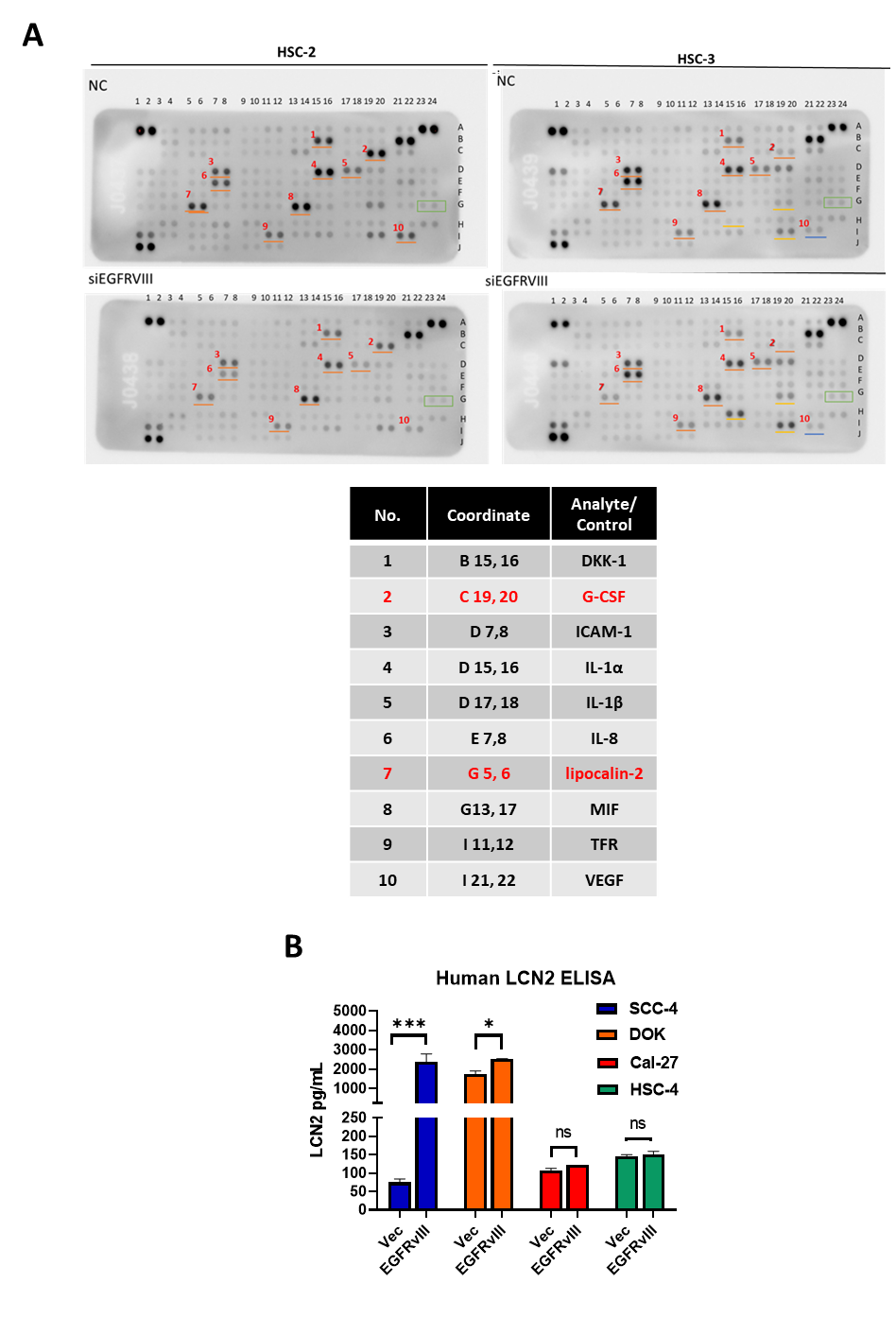
**

**Supplemental Figure 5. Differential expression of cytokines in OSCC cells following EGFRvIII knockdown**

**A.** Proteome analysis using the Proteome Profiler Human XL Cytokine Array Kit with equal amounts of cell lysates from HSC-2 and HSC-3 cells transfected with siEGFRvIII or NC for 72 hours. Significant downregulation of cytokines and regulatory proteins, including LCN2, DKK-1, G-CSF, ICAM-1, IL-1α, IL-1β, IL-8, MIF, and VEGF, was observed in the siEGFRvIII group compared to NC. **B.** ELISA analysis of LCN2 levels in the conditioned medium derived from DOK, Cal-27, HSC-4, and SCC-4 cells transfected with EGFRvIII or control vector for 72 hours. Statistical significance was determined using appropriate tests (*p < 0.05, **p < 0.01, ***p < 0.001).

**Supplemental Figure 6**

**
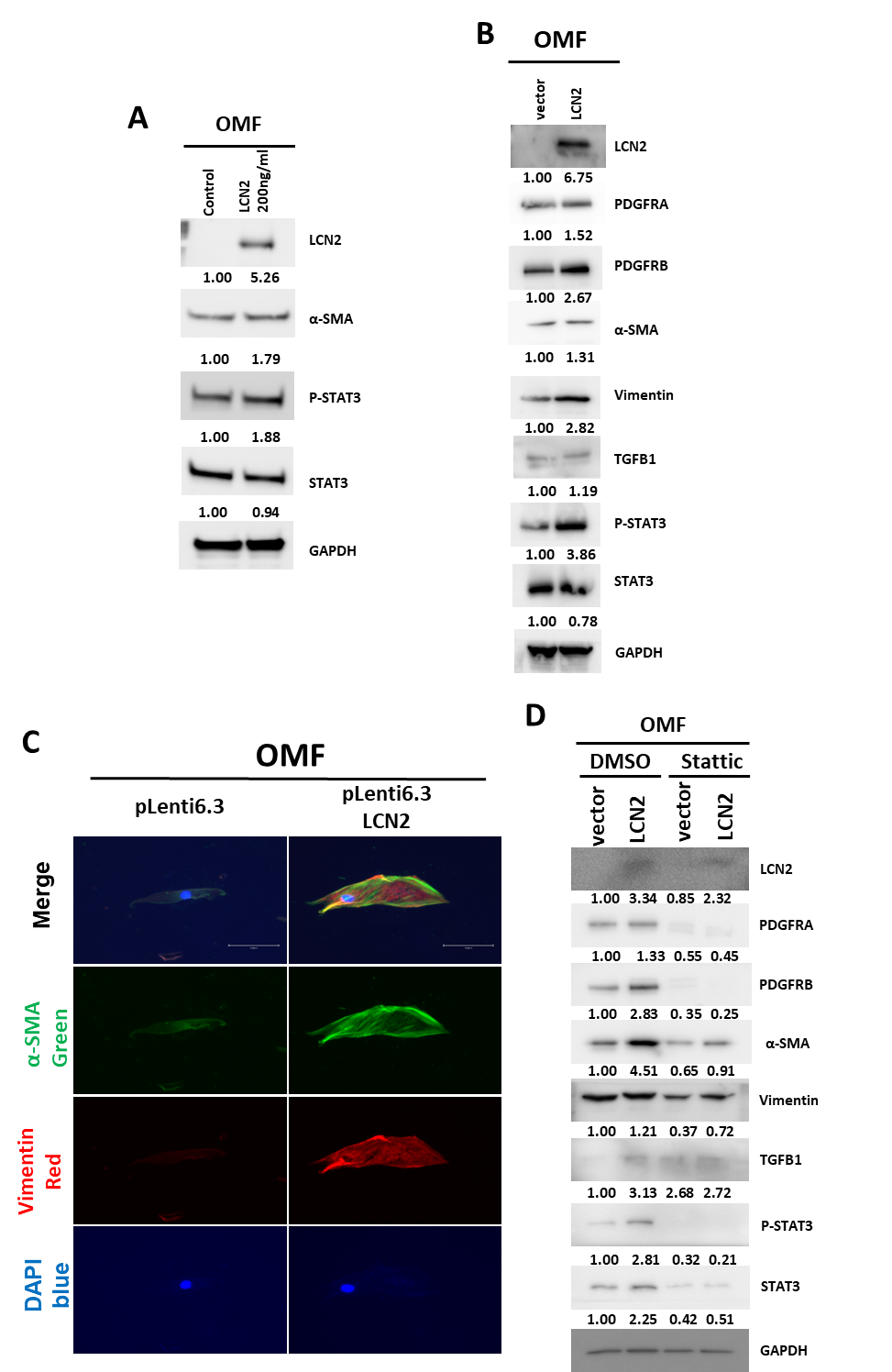
**

**Supplemental Figure 6. Lipocalin-2 induces α-SMA and STAT3 phosphorylation in fibroblasts**

**A.** Western blot analysis of LCN2, α-SMA, STAT3, and phosphorylated STAT3 in OMFs treated with recombinant LCN2 protein (200 ng/mL) or control. Protein band intensities were quantified by densitometry, normalized to GAPDH, and presented as numerical values. **B.** Western blot analysis of LCN2, α-SMA, STAT3, and phosphorylated STAT3 in oral mucosal fibroblasts (OMFs) transfected with LCN2 overexpression vector (OE) or control vector (Vec) for 72 hours. **C.** Immunofluorescence staining of α-SMA (green), Vimentin (red), and nuclei stained with DAPI (blue) in OMFs transfected with LCN2 OE or Vec for 72 hours. Scale bar, 150 μm. **D.** Western blot analysis of fibroblast activation markers (α-SMA, Vimentin, TGFβ1, PDGFRα, PDGFRβ, STAT3, and P-STAT3) in OMFs transfected with an LCN2 overexpression vector or control vector in the presence or absence of Stattic (100 nM, a STAT3 inhibitor) for 72 hours. Protein band intensities were quantified by densitometry, normalized to GAPDH, and presented as numerical values below the gels.

**Supplemental Figure 7**

**
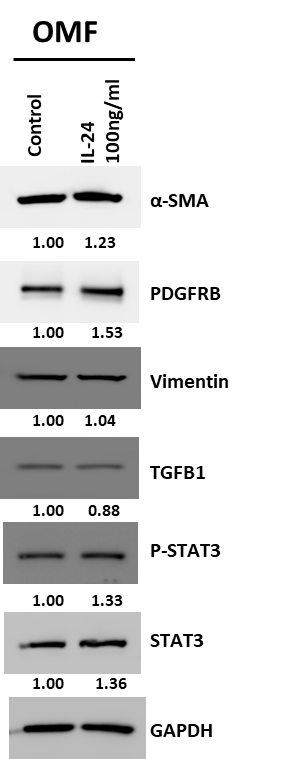
**

**Supplemental Figure 7.**  **IL-24 modestly induces α-SMA expression and STAT3 phosphorylation in fibroblasts.**

Western blot analysis of α-SMA, PDGFRB, Vimentin, TGFB1, total STAT3, and phosphorylated STAT3 (p-STAT3) in oral mucosal fibroblasts (OMFs) treated with recombinant IL-24 protein (100 ng/mL) or vehicle control. Protein band intensities were quantified by densitometry, normalized to GAPDH, and shown as numerical values below each band.

**Supplemental Figure 8**

**
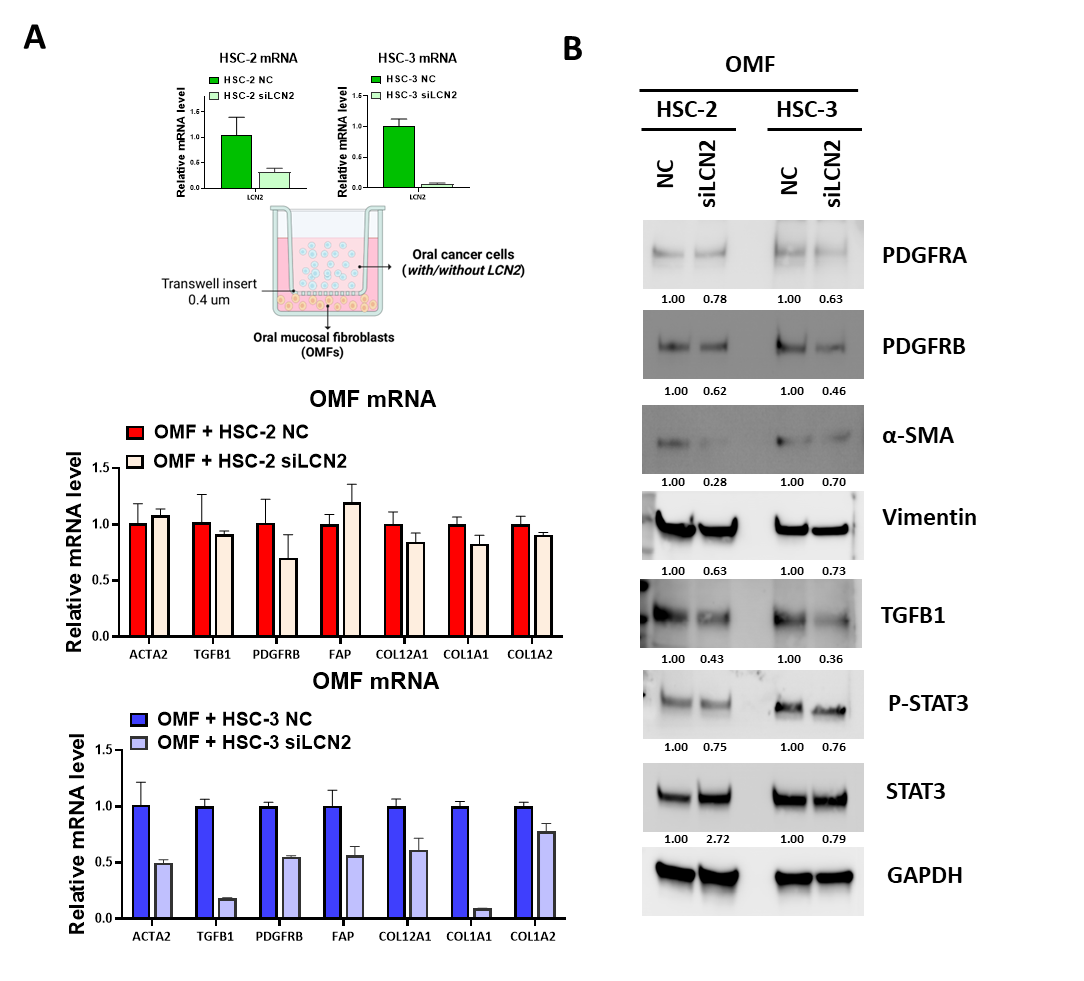
**

**Supplemental Figure 8. Lipocalin-2 (LCN2) knockdown in oral cancer cells suppresses fibroblast activation.**

Oral mucosal fibroblasts (OMFs) were co-cultured with EGFRvIII-overexpressing oral squamous cell carcinoma (OSCC) cells (HSC-2 and HSC-3) that had been transfected with either control siRNA or LCN2-specific siRNA. **A.** Top: Schematic representation of the transwell co-culture system used to study paracrine interactions between OSCC cells and fibroblasts. Bottom: Quantitative PCR analysis of fibroblast activation markers (α-SMA, FAP, TGFβ1, COL12A1, COL1A1, COL1A2, and PDGFRB) in fibroblasts after 72 hours of co-culture with HSC-2 and HSC-3 cells transfected with control or LCN2 siRNA. **B.** Western blot analysis revealed that LCN2 knockdown in OSCC cells significantly reduced the expression of fibroblast activation markers, including α-SMA, PDGFRA, PDGFRB, and vimentin. Furthermore, levels of phosphorylated STAT3 (p-STAT3) were markedly decreased under LCN2-deficient conditions, while total STAT3 and GAPDH served as loading controls. These findings indicate that tumor-derived LCN2 is essential for the paracrine-mediated activation of fibroblasts, and that this effect is at least partially mediated through the STAT3 signaling pathway.

**Supplemental Figure 9**

**
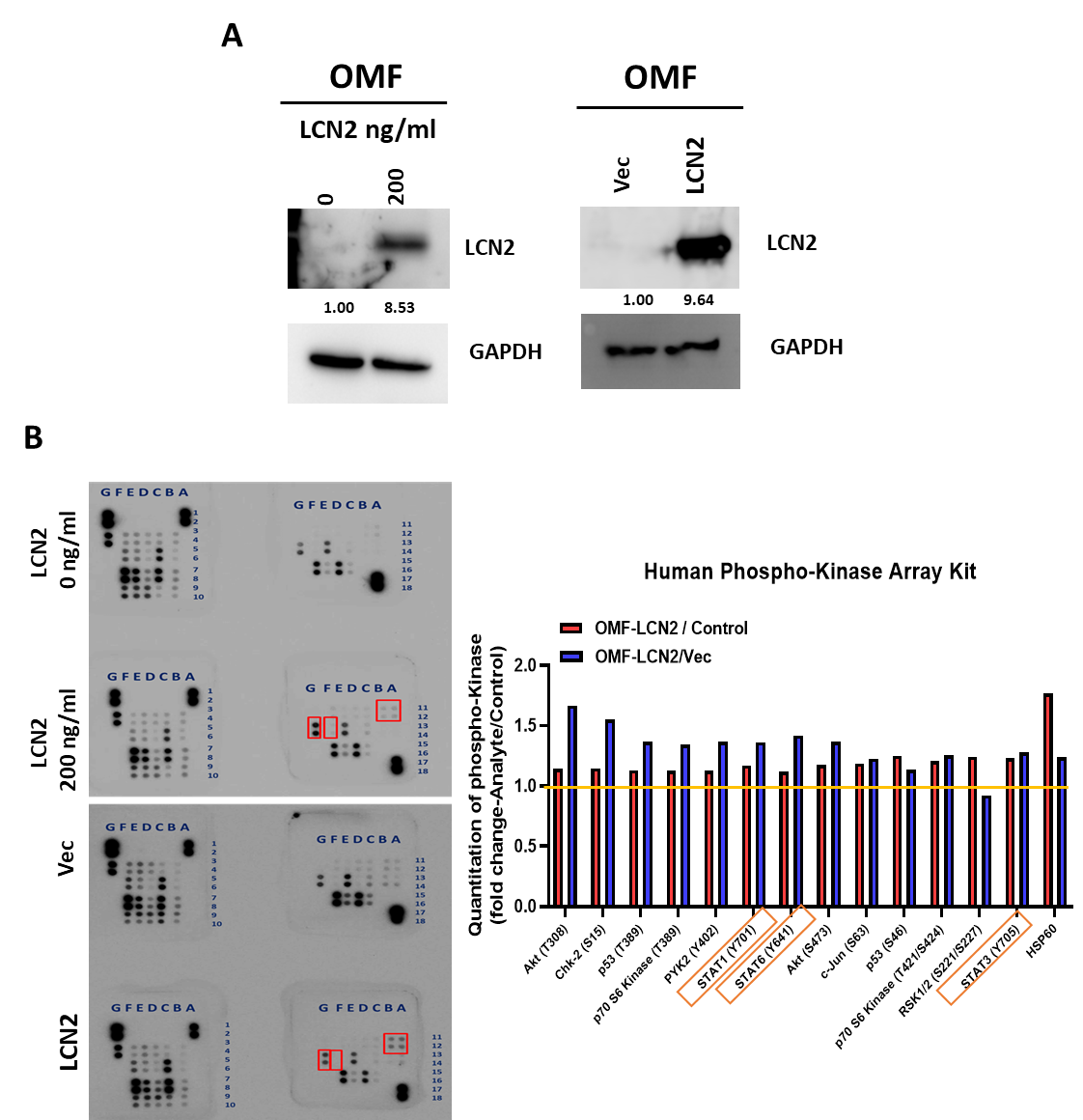
**

**Supplemental Figure 9.** **Lipocalin-2 (LCN2) regulates STAT activation in fibroblasts**

**A.** Western blot analysis of LCN2 protein levels in OMFs treated with LCN2 (0 or 200 ng/mL) or transfected with an LCN2 overexpression vector or control vector for 72 hours. GAPDH was used as a loading control. **B.** Proteome analysis using the Proteome Profiler Human XL Cytokine Array Kit with equal amounts of cell lysates from OMFs treated with LCN2 (0 or 200 ng/mL) or transfected with LCN2 overexpression vector for 72 hours.

**Supplemental Figure 10**

**
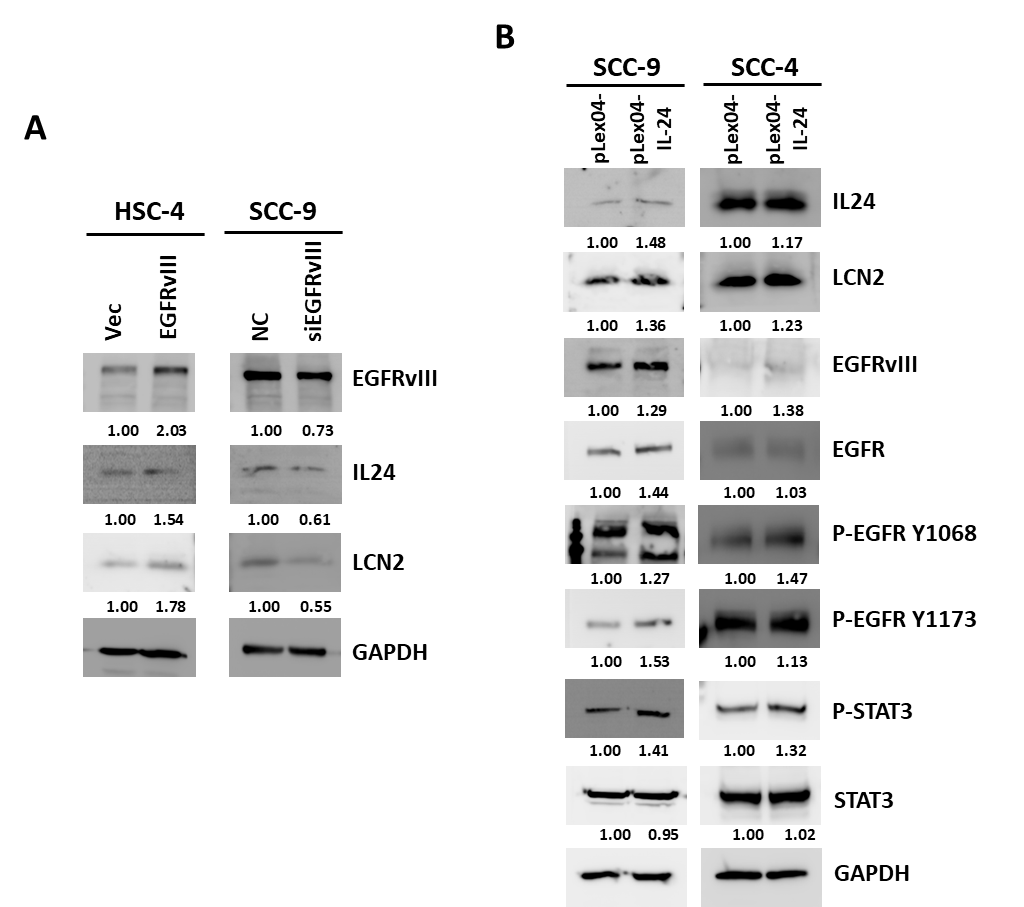
**

**Supplemental Figure 10. IL‐24/EGFRVIII/STAT3 loop in OSCC cells**

**A.** Western blot analysis of EGFRvIII, PD-L1, IL-24, and LCN2 protein levels in HSC-4 cells transfected with EGFRvIII overexpression vector (OE) or control vector (Vec), and SCC-9 cells transfected with siEGFRvIII or non-targeting control (NC) for 72 hours. GAPDH was used as a loading control. **B.** Western blot analysis of EGFRvIII, EGFR, phosphorylated EGFR, PD-L1, IL-24, LCN2, STAT3, and phosphorylated STAT3 protein levels in SCC-9 and SCC-4 cells transfected with IL-24 overexpression vector (OE) or control vector (Vec) for 72 hours. GAPDH was used as a loading control.
